# Biofluid biomarkers for Alzheimer’s disease

**DOI:** 10.3389/fnagi.2024.1380237

**Published:** 2024-04-10

**Authors:** Sensen Wang, Sitan Xie, Qinpin Zheng, Zhihui Zhang, Tian Wang, Guirong Zhang

**Affiliations:** ^1^Shandong Yinfeng Academy of Life Science, Jinan, Shandong, China; ^2^School of Pharmacy, Key Laboratory of Molecular Pharmacology and Drug Evaluation, Ministry of Education, Collaborative Innovation Center of Advanced Drug Delivery System and Biotech Drugs in Universities of Shandong, Yantai University, Yantai, Shandong, China

**Keywords:** Alzheimer’s disease, diagnosis, biofluid, biomarkers, neurodegenerative diseases

## Abstract

Alzheimer’s disease (AD) is a multifactorial neurodegenerative disease, with a complex pathogenesis and an irreversible course. Therefore, the early diagnosis of AD is particularly important for the intervention, prevention, and treatment of the disease. Based on the different pathophysiological mechanisms of AD, the research progress of biofluid biomarkers are classified and reviewed. In the end, the challenges and perspectives of future research are proposed.

## 1 Introduction

Alzheimer’s disease (AD) is the most common type of dementia, which accounts for 60 ~ 80% of all cases (Gauthier et al., 2022). According to the “World Alzheimer’ Report in 2019,” there were about 55 million dementia patients in the world, and this number was expected to reach 152 million by 2055 (Alzheimer’s Disease International, 2019). In 2019, the global societal cost of dementia was around 1.3 trillion US dollars, of which 50% was from the economic value of unpaid care. The 2017 World Health Assembly (WHA) recognized dementia as a public health priority (World Health Organization, 2017). Nowadays, dementia has become one of the biggest public health challenges in the world.

AD can be divided into early-onset AD (EOAD) and late-onset AD (LOAD) at the age boundary of 60 or 65. It could also be classified into familial AD (FAD) and sporadic AD (SAD) based on the family history (Xie et al., 2022). LOAD is mainly SAD, accounting for around 95% of all cases. EOAD is relatively rare, which accounts for less than 5% of AD (Alzheimer’s Disease International, 2019). Jia et al. (2022) reported a 19-year-old AD patient with the memory impairment occurring at the age of 17, which was the youngest probable AD case in the world.

The amyloid plaques and the neurofibrillary tangles (NFT) are the two main pathological characteristics of AD (Blennow et al., 2006; Kang et al., 2022; Mahaman et al., 2022). The amyloid plaques are formed by deposition of extracellular β-amyloid protein (Aβ), and the NFT is induced by intracellular tau hyperphosphorylation. The etiology of AD is still unclear. However, it is generally believed that AD is induced by multiple factors, e.g., genetics, biology, environment, and social psychology (Vermunt et al., 2019; Kulichikhin et al., 2021). The potential mechanism of Aβ-induced neurodegeneration is always the research focus. Human soluble Aβ dimers and trimers induce progressive loss of hippocampal synapses. When exposes to picomolar level of soluble Aβ oligomers, pyramidal neurons in rat brain slice significantly reduce the density of dendritic spines and the number of electrophysiologically active synapses (Shankar et al., 2007). Aβ could directly incorporate into neuronal membranes of hypothalamic neurons, and participate in the formation of calcium-permeable pores, leading to an increase in intracellular calcium concentration of GT1-7 cells. Therefore, the disruption of calcium homeostasis by “Aβ-channels” is recognized as the molecular basis for Aβ neurotoxicity. Previous studies indicated that the lipid composition of cell membrane played an important role in the formation of this channel (Kawahara and Kuroda, 2000). Normally, tau proteins bind to microtubules for maintaining the stability of cytoskeleton. The hyperphosphorylated tau aggregates to form paired helical filaments, which have fewer binding sites and unable to attach to microtubules, thus forming NFT (Liu et al., 2019). NFT disintegrates the microtubule network of nerve cells, resulting in the inhibition of cell biochemical communication, the destruction of the cytoskeleton, and ultimately the production of neurotoxicity (Srivastava et al., 2021). Research data showed that Aβ induced the spread of tau pathology in an unknown way leading to neuronal death (Long and Holtzman, 2019; Karran and De Strooper, 2022). Once Aβ accumulation exceeding a specific threshold, the spread of tau pathology was significantly accelerated (Karran and De Strooper, 2022). However, the hypothesis that the interaction between Aβ and tau leads to cytopathology is still required further investigation.

Since the conceptual framework of preclinical AD was officially proposed by the National Institute of Aging and the Alzheimer’s Association (NIA-AA) in 2011, accumulating data suggested that cognitive decline occurred continuously and progressively over a long period (Jack et al., 2018). For example, the whole course of AD for a 70-year-old person could take approximately 15–25 years, including ~10-year asymptomatic stage (preclinical stage), 4-year mild cognitive impairment, and 6-year for ultimately developing into dementia (Scheltens et al., 2021). Therefore, the measurement of AD biomarker should be a continuous process that begins before symptom appearance (Fleisher et al., 2015; Jack et al., 2018).

Cerebrospinal fluid (CSF) directly reflects pathological changes in brain. Core CSF biomarkers of AD include Aβ_42_, total tau protein (T-tau) and P-tau. Aβ_42_ reflects cortical amyloid deposition. T-tau indicates the density of neurodegeneration, and P-tau links to the pathological changes in NFT. The increased concentration of CSF T-tau and P-tau was found in AD patients (Reitz and Mayeux, 2014), and their diagnostic accuracy was around 85–90% (Visser et al., 2009). However, the CSF or imaging analysis of AD biomarkers is either invasive or expensive (e.g., 10,000 RMB/per time for positron emission tomography (PET), 1,500 RMB/per time for magnetic resonance imaging (MRI), 300 RMB/per time for computed tomography (CT), 1,000–3,000 RMB/per time for CSF examination), or both (Teunissen et al., 2022).

Based on the characteristics of accessibility, sampling technology, repeatability and cost-effectiveness, blood biomarkers have more advantages than CSF and imaging analysis (Blennow and Zetterberg, 2018). However, the detection of AD biomarkers in blood is much more complex than in CSF (Kulichikhin et al., 2021). First, there is a blood–brain barrier (BBB) in the human body. The capillary endothelium in BBB lacks pores (Ueno et al., 2016), and therefore ions and polar molecules could only cross the BBB in the presence of some transport proteins (Haas, 2018). However, the transporters that are responsible for transporting tau through BBB have not yet been identified (Ueno et al., 2016). When the axon is damaged, proteins are released from the extracellular space of the brain and only a small fraction could enter the bloodstream. These brain proteins are cleaved, modified, and degraded before or after passing through BBB (Kulichikhin et al., 2021). Second, the blood-cerebrospinal fluid barrier (BCB) is another important barrier. BCB is porous, so small peptides and hydrophilic molecules could pass through it. Due to the death of neuronal cells and intracellular high concentration, tau is firstly released to CSF (Tarasoff-Conway et al., 2015), and then enters the blood through the barrier (Haas, 2018). Because of the existence of BBB and BCB, there is a difference on the concentration between CSF and blood biomarkers. Third, plasma contains multiple background proteins. Some of them are at high levels. Therefore, the analytical techniques of blood samples should have a high sensitivity and specificity in order to detect a small quantity of biomarkers in the complex backgrounds (Kulichikhin et al., 2021).

**Figure 1:**
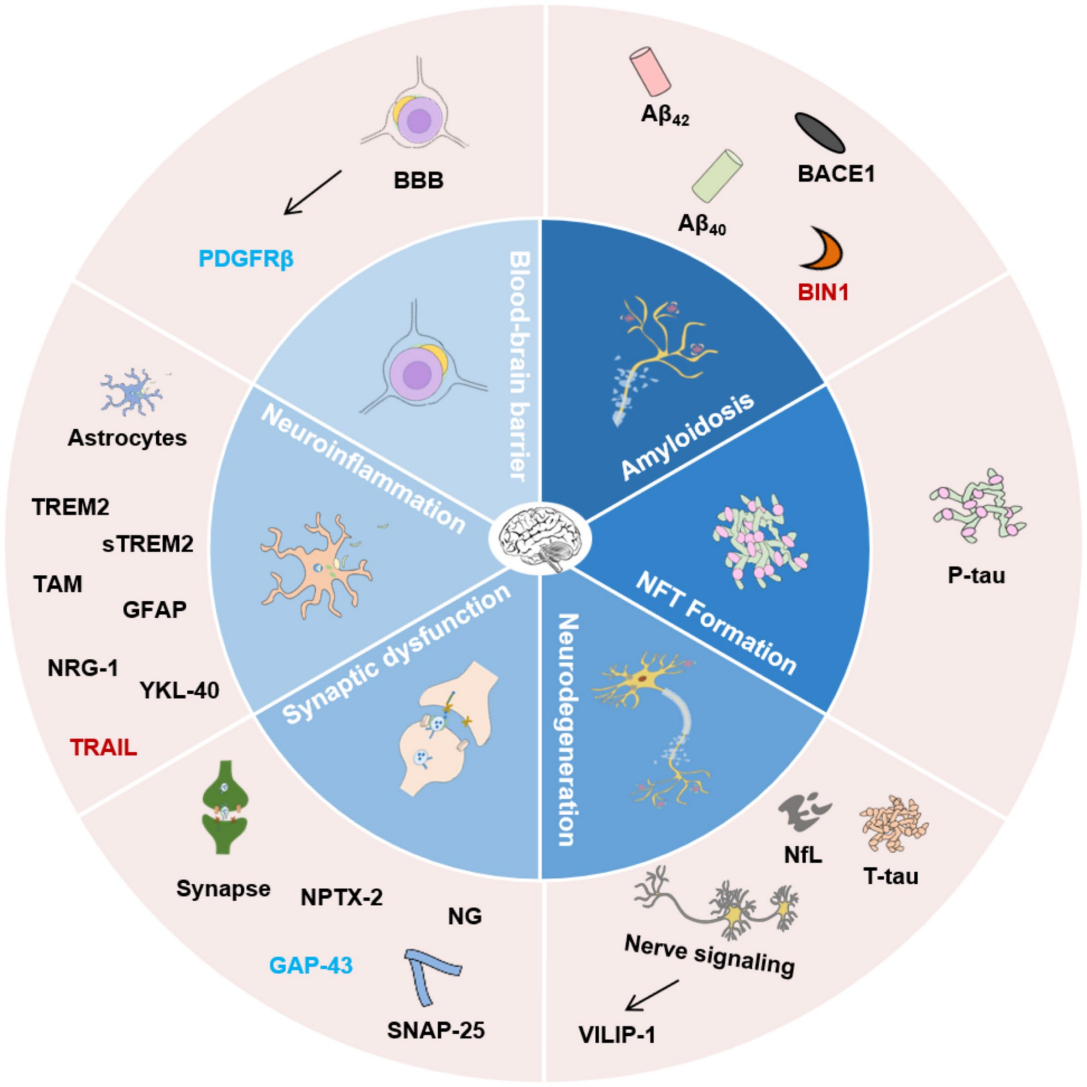
Classification of AD biofluid biomarkers described in this review based on different pathophysiological mechanisms. The inner ring indicates different potential mechanisms. The outer ring lists the biomarkers. Biomarkers with different colors present the corresponding research in different biological matrices. Black, CSF and blood; Blue, CSF; Red, blood. Aβ_42_, β-amyloid 42; Aβ_40_, β-amyloid 40; BACE1, β-secretase enzyme; BIN1, Bridging integrator 1; NfL, Neurofilament Light Chain; NFT, Neurofibrillary tangles; VILIP-1, Visinin-like Protein 1; SNAP-25, Synaptosome-associated protein 25; NPTX-2, Neuronal pentraxin 2; GAP-43, Growth-associated protein 43; NG, Neurogranin; TREM2, Triggering receptor 2; sTREM2, Soluble Triggering receptor 2; GFAP, Glial fibrillary acidic protein; NRG-1, Neuregulin-1; YKL-40, Human cartilage glycoprotein-39; TRAIL, TNF-related apoptosis-inducing ligand; PDGFRβ, Platelet-derived growth factor receptor-β; BBB, Blood–brain barrier.

In this review, the traditional and emerging AD biomarkers are summarized and categorized according to main AD pathologies such as amyloidosis, NFT, neurodegeneration, synaptic dysfunction, neuroinflammation, and BBB breakdown (Figure 1). The physiological function and the biofluid level of these markers are described. In addition, the research of some biomarkers on the other neurodegenerative diseases are also reviewed in this article.

## 2 Biomarkers of amyloidosis

### 2.1 Aβ_42_

Aβ_40_ and Aβ_42_ are the most common subtypes in human. Due to the expanded C-terminal, Aβ_42_ is highly hydrophobic and easier to aggregate than Aβ_40_ (Long and Holtzman, 2019). The level of CSF Aβ_42_ in preclinical stage, mild cognitive impairment (MCI), and AD with dementia symptoms was lower than those in control groups (Bateman et al., 2012). It could decrease to around 50% of the healthy individuals (Table 1; Olsson et al., 2016). A neuropathological examination showed that a decrease in CSF Aβ_42_ was associated with an increase in brain amyloid plaques (Strozyk et al., 2003). Elderly persons with a decrease of CSF Aβ_42_ were also Aβ-PET positive, and vice versa (Fagan et al., 2006). The studies indicated that a reduction in CSF Aβ_42_ preceded the formation of plaques (Palmqvist et al., 2016; Kulichikhin et al., 2021). Thus, CSF Aβ_42_ is considered as a robust biomarker of early AD diagnosis. A decrease in CSF Aβ_42_, coupled with an increase in T-tau and P-tau could help to identify the symptomatic AD (Blennow and Hampel, 2003). Low level of CSF Aβ_42_, rather than high level of T-tau, could predict cognitive decline (Stomrud et al., 2007). In addition, a significant decrease in CSF Aβ_42_ was observed in Creutzfeldt-Jakob disease (CJD), multiple system atrophy (MSA), and amyotrophic lateral sclerosis (ALS) (Holmberg et al., 2003), which indicated that CSF Aβ_42_ could be influenced by other factors besides plaque formation (Mahaman et al., 2022).

**Table 1:** Summarized information of AD amyloidosis biofluid biomarkers presented in this review.

Pathophysiological mechanism	Biomarker	Biological matrices	Trend	Purpose
Amyloidosis	Aβ_42_	CSF	Decrease (Olsson et al., 2016)	Diagnosis (Jack et al., 2018)
		Blood	Controversial (Mayeux et al., 2003; Nakamura et al., 2018)	Research (Wang J. et al., 2018)
	Aβ_40_	CSF	No significant change (Kulichikhin et al., 2021)	Research (Janelidze et al., 2016b; Kulichikhin et al., 2021)
		Blood	Decrease (Janelidze et al., 2016b)	
	Aβ_42_/Aβ_40_	CSF	Decrease (Schindler et al., 2019)	Diagnosis (Jack et al., 2018)
		Blood	Decrease (Janelidze et al., 2016b)	Research (Schindler et al., 2019)
	T-tau/Aβ_42_	CSF	Increase (Kaplow et al., 2020)	Research (Kaplow et al., 2020)
	P-tau181/Aβ_42_	CSF		
	APP669-711/Aβ_42_	Blood	Increase (Nakamura et al., 2018)	Research (Nakamura et al., 2018)
	BACE1	CSF	Increase (Cervellati et al., 2020)	Research (Decourt and Sabbagh, 2011)
		Blood		
	BIN1	Blood	Increase (Sun et al., 2013)	Research (Sun et al., 2013)

Plasma Aβ analysis is one of the most widely applied peripheral biomarker tests of AD (Mahaman et al., 2022). Plasma and CSF Aβ_42_ had a weak positive correlation, while plasma Aβ_42_ levels and brain Aβ deposition had a negative correlation (Janelidze et al., 2016b). The research results regarding the relationship between plasma Aβ_42_ and cognitive impairment were not consistent. Some studies showed decreased plasma Aβ_42_ in MCI and AD (Nakamura et al., 2018), while others reported increased Aβ_42_ level and following decreased trend before or at the beginning of cognitive decline (Mayeux et al., 2003). The inconsistent results are probably caused by differences in sample inclusion and exclusion criteria, as well as the analytical methods (Wang J. et al., 2018). Elevated levels of plasma Aβ_42_ and Aβ_40_ were also associated with other diseases, such as hypertension, diabetes and ischemic heart disease, indicating significant differences in metabolic process of amyloid proteins between blood and brain (Janelidze et al., 2016b). Despite these differences, all studies confirmed the changes of blood amyloid proteins at the early stages of AD. In the future, the association between AD pathology and blood amyloid protein is required further exploration to promote its clinical application.

### 2.2 Aβ_40_

Aβ_40_ is the most abundant protein fragment hydrolyzed from APP. The concentration of CSF Aβ_40_ had no significant difference during the development of AD (Kulichikhin et al., 2021). Similar to Aβ_42_, there was a weak positive correlation between plasma and CSF Aβ_40_ (Janelidze et al., 2016b). Although Aβ_40_ does not have the same strong cytotoxicity as Aβ_42_, Aβ_40_ aggregates could be detected in cerebral amyloid vasculopathy (Attems et al., 2011). The decreased plasma concentration of Aβ_40_ was observed in AD patients compared with controls (Janelidze et al., 2016b). Biochemical and molecular simulation showed that Aβ_40_ inhibited the aggregation of Aβ_42_ (Jan et al., 2008).

### 2.3 Aβ_42_/Aβ_40_

The impact of total Aβ variation could be neutralized through normalization of Aβ_42_ by Aβ_40_ (Lewczuk et al., 2004). Several studies showed that CSF Aβ_42_/Aβ_40_ could better diagnose, differentiate, and monitor AD than CSF Aβ_42_ (Hansson et al., 2019). CSF Aβ_42_/Aβ_40_ and Aβ-PET positive had a good correlation (Kulichikhin et al., 2021). Compared to negative Aβ-PET population, the CSF Aβ_42_/Aβ_40_ level of positive Aβ-PET individuals decreased (Schindler et al., 2019). PD and dementia with Lewy body (DLB) had higher level of CSF Aβ_40_. Therefore, Aβ_42_/Aβ_40_ could help to distinguish AD from these diseases (Nutu et al., 2013; Mahaman et al., 2022). The combined application of CSF Aβ_42_/Aβ_40_ and other CSF biomarkers, e.g., T-tau or P-tau, gave a better prediction of AD (Baldeiras et al., 2018) and the conversion from MCI to AD (Baldeiras et al., 2018; Mahaman et al., 2022). Thus, CSF Aβ_42_/Aβ_40_ is proposed as a promising biomarker for preclinical AD diagnosis (Hansson et al., 2019; Kulichikhin et al., 2021).

In a 719-person cohort study, the plasma concentration of Aβ_42_/Aβ_40_ significantly decreased in MCI and AD patients (Janelidze et al., 2016b). Ovod et al. (2017) reported that plasma Aβ_42_/Aβ_40_ was decreased in patients with cerebral amyloidosis. Cognitive decline and the risk of AD progression were associated with low plasma Aβ_42_/Aβ_40_ levels (Verberk et al., 2020). Plasma Aβ_42_ alone was not an accurate biomarker for AD brain pathology, while Aβ_42_/Aβ_40_ could give a better prediction (Ovod et al., 2017; Schindler et al., 2019). Moreover, plasma Aβ_42_/Aβ_40_ was associated with Aβ-PET (Schindler et al., 2019). Cognitively normal individuals with declined Aβ_42_/Aβ_40_ were observed within Aβ-PET-negative groups, indicating the early stage of AD before plaque formation (Schindler et al., 2019). A mathematical simulation study suggested that the risk of positive Aβ-PET for the above population was 15 times higher than those with normal plasma Aβ_42_/Aβ_40_ in the next 6 years (Schindler et al., 2019). Additionally, plasma Aβ_42_/Aβ_40_ and Aβ_42_/Aβ_38_ had higher accuracy than Aβ_42_ in distinguishing AD from DLB, PD, or subcortical vascular dementia (VaD) (Janelidze et al., 2016c).

### 2.4 Other Aβ ratios

Elevated CSF T-tau/Aβ_42_ or P-tau181/Aβ_42_ indicated an obvious AD brain pathology (Kaplow et al., 2020). CSF Aβ_42_/P-tau181 showed high accuracy in predicting the progression from MCI to AD (Buchhave et al., 2012; Mahaman et al., 2022). A study based on mass spectrometry suggested that the plasma levels of APP669-711/Aβ_42_ and Aβ_40_/Aβ_42_ in Aβ-PET-positive individuals were higher than those with negative Aβ-PET scan (Nakamura et al., 2018). These two ratios were associated with the levels of CSF Aβ_42_ (Kaneko et al., 2014; Nakamura et al., 2018), and they could be used to predict the brain Aβ burden (Nakamura et al., 2018).

### 2.5 BACE1

BACE1, also known as β-secretase or β-site APP-lyase 1, is encoded by *BACE1* gene, which is primarily expressed in neurons of brain and responsible for Aβ production (Vassar et al., 1999). It was reported that the increase of *BACE1* gene expression or the abnormal function of β-secretase was one of the earliest processes in AD (Yang et al., 2003). Knocking out *BACE1* in mice resulted in a significant decrease in Aβ and CTFβ concentration (a 99-amino acid fragment starting with the N-terminal aspartic acid residue of Aβ) in the brain (McConlogue et al., 2007). In the SAD brain, the expression of BACE1 significantly increased (Yang et al., 2003). The CSF level of BACE1 protein of AD patients were significantly elevated (Cervellati et al., 2020). The activity of CSF BACE1 was higher in the individuals with the conversion from MCI to AD than those without (Zetterberg et al., 2008). A recent research (Hampel et al., 2021) and two large-scale cohort studies (Zuliani et al., 2020) presented similar trend of CSF BACE1 in peripheral blood, with a 30% increase in serum of AD compared to the control group. In *APP/PS-1* transgenic mice, abnormal BACE1 expression in the retina predated behavioral defects. Therefore, BACE1 could be used as a sensitive biomarker for the early diagnosis of AD (Decourt and Sabbagh, 2011; Haas, 2018). However, the application of CSF BACE1 as a clinical AD biomarker is limited by its inter-subject variability and technical difference of assay in the lab (Hampel et al., 2020). The research focus is mainly on the *BACE1* gene rather than the level of BACE1 protein in the other neurodegenerative diseases such as PD (Li et al., 2020).

### 2.6 BIN1

Bridging integrator 1 (BIN1) is a member of amphiphysin proteins family. It is associated with the cytoskeleton and cell membrane. BIN1 plays an important role in the nervous system (Sudwarts et al., 2022). BIN1 is widely expressed in mice and human brain (Miyagawa et al., 2016). It participates in the regulation of endocytose (Wechsler-Reya et al., 1998), and it is important in intracellular vesicles sorting (Pant et al., 2009). Previous study showed that BIN1 affected the AD molecular pathobiology through the regulation membrane trafficking of AD-related proteins (Miyagawa et al., 2016). BIN1 dependent pathophysiological process is possibly associated with tau pathology through various mechanisms (Thomas et al., 2019). However, there is no further evidence to clarify whether this effect is regulated through the modulation of tau phosphorylation (Thomas et al., 2019), the influence of BIN1-tau interactions (Malki et al., 2017; Sartori et al., 2019), or directly facilitating the propagation of pathogenic tau (Crotti et al., 2019). Genome-wide association studies demonstrated that *BIN1* was a genetic risk factor of LOAD (Tan et al., 2013). The depletion of *BIN1* enhanced the level of cellular BACE1 by impairing endosomal trafficking and decreased BACE1 lysosomal degradation, leading to the overproduction of Aβ (Miyagawa et al., 2016). A cohort study including 112 AD and 200 control subjects showed significantly elevated levels of BIN1 mRNA and protein in the plasma of ADs. However, this trend is required further investigation in the larger-scale cohort (Sun et al., 2013).

## 3 Biomarkers of NFT formation

### 3.1 P-tau

AD-related NFT are composed of P-tau (Grundke-Iqbal et al., 1986). CSF P-tau was elevated at the preclinical stage of AD (Sato et al., 2018), and it continued to rise during the early clinical stage (Palmqvist et al., 2019). Higher CSF T-tau and P-tau indicated faster disease progression (Wallin et al., 2010). The elevated CSF T-tau reflected CNS degeneration, while increased P-tau was specific to AD (Blennow and Hampel, 2003; Skillbäck et al., 2014). Therefore, P-tau could help to distinguish AD from other neurodegenerative diseases (Hampel et al., 2004). The correlation between CSF P-tau and Aβ was stronger than that between CSF P-tau and tau-PET (La Joie et al., 2018). This is probably because Aβ pathology is the reason for the increased CSF P-tau, while tau-PET measures NFT (Sato et al., 2018; Smirnov et al., 2022).

Several CSF P-tau proteins were reported to increase at the preclinical stage of AD. Both P-tau181 and P-tau217 began to increase at the early stage of AD, which were around 20 years before the detection of tau aggregation in the brain (Barthélemy et al., 2020b). Compared to the control groups, the CSF P-tau217 increased by 5 times in AD patients, while P-tau181 only increased by 1.3 times (Barthélemy et al., 2019). CSF or plasma P-tau217 could identify patients with Aβ-PET positive but tau-PET negative, which indicated the changes of CSF P-tau before the detection of tau aggregates (Barthélemy et al., 2020a; Kulichikhin et al., 2021). CSF P-tau205 increased in the advanced AD, and it was related to the increase of T-tau and brain atrophy (Barthélemy et al., 2020b). Studies reported that P-tau231 was the earliest increasing biomarker in AD (Ashton et al., 2021; Smirnov et al., 2022). There are few studies about comparison of different CSF P-tau biomarkers. One study showed that the levels of CSF P-tau181, P-tau199 and P-tau231 were strongly associated with each other (Hampel et al., 2004). By combining the application of two or more biomarkers, higher diagnostic accuracy could be obtained (Hansson et al., 2006). The fragments of the microtubule binding region (MTBR) where tau was much easier to aggregate (Blennow et al., 2020) were more reliable indicators for tau aggregation (Simrén et al., 2023). A mass spectrometry study investigating the role of MTBR showed that CSF MTBR-tau, such as MTBR-tau243, MTBR-tau299 or MTBR-tau354, was a promising biomarker for monitoring target participation in the clinical trials (Horie et al., 2021).

Plasma P-tau was suggested to be the most promising analyte as a screening tool in clinical application. The increase of plasma P-tau was closely related to amyloid plaques and tau tangles (Mattsson-Carlgren et al., 2021). The validity of plasma P-tau as AD biomarker has been verified in neuropathologically confirmed cases (Lantero Rodriguez et al., 2020; Palmqvist et al., 2020), which was not affected by common co-pathologies (such as TDP-43 or α-synuclein pathology) (Smirnov et al., 2022). Studies proved that plasma P-tau could predict the progression from cognitively unimpaired individuals to MCI and eventually to AD (Janelidze et al., 2020). Plasma P-tau had excellent accuracy in distinguishing AD from non-AD tau proteinosis (e.g., progressive supranuclear paralysis or cortical basal degeneration) or non-AD neurodegenerative diseases [e.g., DLB or frontotemporal dementia (FTD)] (Palmqvist et al., 2020; Ashton et al., 2021; Thijssen et al., 2021). Several P-tau proteins such as P-tau181, P-tau217 and P-tau231 have been proposed as AD blood biomarkers (Palmqvist et al., 2020; Ashton et al., 2021; Smirnov et al., 2022). With the progression of the disease, plasma P-tau181 increased steadily (Suárez-Calvet et al., 2020; Thijssen et al., 2020), and it could accurately distinguish AD from non-AD neurodegenerative diseases (Janelidze et al., 2020). Plasma P-tau 217 was at a high level before detection of tau pathology by PET.

The concentrations of P-tau181, T-tau and Aβ_42_ in exosomes of AD were associated with the corresponding CSF biomarkers. They were significantly higher than those in MCI and healthy controls (Jia et al., 2019). Compared to the control groups, P-tau, Aβ_42_ and phosphorylated insulin receptor substrate 1 (IRS-1) of neuronal derived extracellular vesicles (NDEVs) in ADs showed high accuracy in predicting and distinguishing AD (Kapogiannis et al., 2019).

## 4 Biomarkers of neurodegeneration

In 2018, NIA-AA guideline proposed neurodegeneration as the third biomarker of AD (Table 2; Jack et al., 2018). Although neurodegeneration alone was not enough as a diagnostic marker, its change rate could accurately predict the progression of the disease (Frisoni et al., 2010).

**Table 2:** Summarized information of AD neurodegeneration biofluid biomarkers presented in this review.

Pathophysiological mechanism	Biomarker	Biological matrices	Trend	Purpose
Neurodegeneration	T-tau	CSF	Increase (Shui et al., 2018)	Diagnosis (Jack et al., 2018)
		Blood	Increase (Chen et al., 2019)	Research (Mahaman et al., 2022)
	NfL	CSF	Increase (Zetterberg et al., 2016)	Research (Mattsson et al., 2017; Bridel et al., 2019)
		Blood	Increase (Mattsson et al., 2017)	
	VILIP-1	CSF	Increase (Braunewell, 2012; Halbgebauer et al., 2022b)	Research (Mroczko et al., 2015; Halbgebauer et al., 2022b)
		Blood		

### 4.1 T-tau

In AD patients, the level of CSF T-tau increased (Shui et al., 2018). Similar to CSF P-tau181, CSF T-tau began to increase after the formation of amyloid plaques (Schindler, 2022). This happened 10 ~ 15 years earlier before NFT (Bateman et al., 2012; Fleisher et al., 2015). Therefore, CSF T-tau and P-tau181 probably reflected neuron dysfunction caused by Aβ plaques, rather than tau tangles. The level of CSF T-tau also increased in other diseases, such as stroke, acute neuronal injury, and CJD (Skillbäck et al., 2014). It increased within a few days after acute brain injury, and continued to elevate for several weeks until it finally fell back to the normal range (Zetterberg et al., 2006). Previous studies reported that the highest level of CSF T-tau was observed in the most serious neurodegenerative diseases. For example, CSF T-tau level in CJD patients was 10 to 20 times higher than that in AD (Skillbäck et al., 2014).

A two-year longitudinal study involving 152 people showed that plasma T-tau and T-tau/Aβ_42_ could predict the deposition of abnormally folded proteins in the brain (Sutphen et al., 2018). There was a correlation between high plasma T-tau and AD (Chen et al., 2019). Although the plasma T-tau concentration in MCI or AD increased, the research data showed that the range of plasma T-tau in AD overlapped with that in normal population. This hinders the application of plasma T-tau as a diagnostic biomarker (Mahaman et al., 2022).

### 4.2 Neurofilament light chain (NfL)

Neurofilament plays an important role in maintaining the neuronal integrity and regulating the transport of intracellular components (Liu et al., 2004; Wagner et al., 2007; Yan et al., 2007; Bruno et al., 2012). Because of the imbalance of tau kinase and phosphatase activity, abnormal hyperphosphorylation of neurofilaments occurred leading to the loss of their stability and axonal injury in the white matter and brain (Gong et al., 2000). Axonal injury released neurofilament proteins into interstitial fluid, and they could be detected in CSF (Bruno et al., 2012). The concentration of NfL in CSF rose at the early clinical stages of AD, correlating with cognitive decline and the changes of brain structure (Zetterberg et al., 2016). A longitudinal study involving 389 subjects showed the elevated CSF NfL level in ADs than healthy controls (Mattsson et al., 2016). The lack of correlation between increased CSF NfL and Aβ pathology indicated that NfL was not specific to AD. Higher concentration of CSF NfL indicated the existence of axonal injury (Bridel et al., 2019). Although CSF NfL was not a specific biomarker for a disease, it could help clinicians to investigate whether cognitively impaired patients had neurological causes (Schindler, 2022). Studies showed that combined application of NfL with other biomarkers could greatly improve the accuracy of dementia diagnosis (de Jong et al., 2007; Mattsson et al., 2016). For example, the combination of CSF Aβ_42_ and P-tau181 with CSF NfL improved the diagnosis accuracy of early AD and FTD (de Jong et al., 2007).

Blood NfL was closely related to CSF NfL (Hansson et al., 2017), and it has become an easy-to-obtain biomarker for reflecting the intensity of neuronal damage and neurodegeneration (Simrén et al., 2023). Some studies proposed that blood NfL was an effective tool for the early diagnosis of AD (Fortea et al., 2018). Elevated plasma NfL concentrations were observed in SAD (Ashton et al., 2021). Compared to the healthy controls, the plasma NfL level of AD increased by nearly 150% (Mattsson et al., 2017). The increased NFL was related to the cognitive decline, brain atrophy and low metabolism (Mattsson et al., 2017). Weston et al. pointed out that serum NfL increased in asymptomatic FAD, and it was associated with the stage and severity of the disease (Weston et al., 2017). Due to the concomitant pathologies of axons and peripheral axons, big difference in plasma NfL levels among healthy elderly population limited its specificity (Simrén et al., 2023). Nevertheless, the plasma NfL was more specific in autosomal dominant AD (ADAD) and Down syndrome because of the younger onset age of these disease process (Simrén et al., 2023).

### 4.3 VILIP-1

Visinin-like Protein 1 (VILIP-1) is a new biomarker for reflecting the pathological changes of AD. It is a calcium-binding protein of neuronal calcium sensor (NCS), which plays an important role in neuronal signaling cascades (Burgoyne and Weiss, 2001). In AD and other neurodegenerative diseases, neurons showed abnormal Ca^2+^ homeostasis, which resulted in the aberrant regulation of Ca^2+^ ion channels and reduction of Ca^2+^ buffering capacity (Marambaud et al., 2009). VILIP-1 participated in calcium-mediated neuronal injury through regulation of Ca^2+^ ions. The expression of VILIP-1 was closely related to Aβ plaque and NFT in ADs (Braunewell et al., 2001; Braunewell, 2012). CSF VILIP-1 could predict the change rate of global and focal brain atrophy which was similar as T-tau and P-tau181 (Luo et al., 2013). The CSF VILIP-1 level of *ApoE ε4* carriers was significantly increased, and it was positively correlated with tau and P-tau levels (Wang et al., 2020). A meta-analysis indicated enhanced levels of CSF VILIP-1 in AD compared to the control group. In addition, the CSF VILIP-1 level was higher in MCI patients who progressed to AD than stable MCIs (Mavroudis I. A. et al., 2021). Similarly, several longitudinal studies pointed out that the concentration of VILIP-1 and/or VILIP-1/Aβ_42_ in CSF could be applied to diagnose diseases at an early stage, and predicted the future cognitive impairment of normal individuals (Luo et al., 2013). For example, the VILIP-1/Aβ_42_ and VILIP-1 in the CSF could help to differentiate AD from CJD (Halbgebauer et al., 2022b). No significant difference of VILIP-1 was observed between AD and DLB (Mavroudis I. A. et al., 2021). Serum VILIP-1 could not discriminate AD from PD, ALS, and behavioral variant frontotemporal dementia (bvFTD) (Halbgebauer et al., 2022b). It was reported that serum VILIP-1 was correlated with acute ischemic stroke (Stejskal et al., 2011; Liu D. et al., 2020) and the neuronal injury caused by epileptic seizures (Tan et al., 2020). To date, there is no convincing data supporting the correlation between plasma VILIP-1 and AD or other neurodegenerative diseases.

## 5 Biomarkers of synaptic dysfunction

Synaptic dysfunction is closely correlated with the loss of synaptic integrity and neuropathological changes in the brain, which is another important pathophysiological mechanism of cognitive decline in AD. Synaptic dysfunction is largely driven by Aβ and tau pathology, and/or by the indirect consequence of neuroinflammatory response (Jackson et al., 2019). Therefore, synaptic alterations are considered as an early pathological event of AD (Scheff et al., 2007). Biomarkers indicating synaptic integrity and plasticity are helpful for early diagnosis and prognosis monitoring of AD (Table 3; Marambaud et al., 2009).

**Table 3:** Summarized information of AD synaptic dysfunction biofluid biomarkers presented in this review.

Pathophysiological mechanism	Biomarker	Biological matrices	Trend	Purpose
Synaptic dysfunction	SNAP-25	CSF	Increase (Öhrfelt et al., 2016)	Research (Öhrfelt et al., 2016; Agliardi et al., 2019)
		Blood NDEs	Decrease (Agliardi et al., 2019)	
	GAP-43	CSF	Increase (Sandelius et al., 2019)	Research (Sandelius et al., 2019)
	NPTX-2	CSF	Decrease (Goetzl et al., 2018; Nilsson et al., 2021)	Research (Goetzl et al., 2018; Nilsson et al., 2021)
		Blood NDEs		
	NG	CSF	Increase (Portelius et al., 2018)	Research (Liu W. et al., 2020)
		Blood exosome	Decrease (Liu W. et al., 2020)	

### 5.1 Presynaptic protein

Synaptosome-associated protein 25 (SNAP-25) exists in synaptic vesicles (Geppert et al., 1994). CSF SNAP-25 is a key participant in synaptic degeneration (Zhang et al., 2018). An increasing trend of CSF SNAP-25 was observed in the AD patients (Öhrfelt et al., 2016). The significantly reduced SNAP-25 in the cerebral cortex indicated the synaptic dysfunction (Davidsson and Blennow, 1998). It was reported that CSF SNAP-25 could differentiate AD from PD, FTD and ALS, and high concentration of CSF SNAP-25 could be applied as a biomarker for both AD and CJD (Halbgebauer et al., 2022a). CSF SNAP-25 level was correlated with *APOE ε4* (Wang S. et al., 2018). MCI patients carrying *APOE ε4* showed higher CSF SNAP-25 than non-carriers (Wang S. et al., 2018). There is few research on the correlation between blood SNAP-25 and AD. A study presented the reduced SNAP-25 in the neuron-derived exosomes (NDEs) isolated from serum, and it was associated with the cognition evaluated by Mini-Mental State Examination (MMSE) (Agliardi et al., 2019).

The increase of growth-associated protein 43 (GAP-43) was considered as another biomarker reflecting AD-related synaptic dysfunction. CSF GAP-43 increased with the cognitive decline, and it was correlated with Aβ plaque and NFT in hippocampus, amygdala, and cortex (Sandelius et al., 2019). CSF GAP-43 could predict the progression from MCI to AD, and this correlation was suspected to be related with *APOE ε4* (Zhu et al., 2023). Elevated level of CSF GAP-43 was specific to AD compared to the other neurodegenerative diseases, e.g., MCI, ALS, behavioral variant FTD (bvFTD), PD, DLB, primary progressive aphasia (PPA), progressive supranuclear palsy, corticobasal syndrome, and posterior cortical atrophy (PCA). Therefore, it was considered as a promise AD biomarker for the clinical research. However, another study reported a temporary increase of CSF GAP-43 after ischemic stroke (Sandelius et al., 2018). A blood neuro-exosomal study presented that exosomal GAP-43 could predict MCI 5–7 years in advance (Jia et al., 2021).

Neuronal pentraxin 2 (NPTX-2) is a protein correlated with the inhibitory circuit dysfunction. NPTX-2 showed different trends from the above biomarkers. The level of NPTX-2 in AD cerebral cortex and CSF decreased, and it had a strong correlation with cognitive ability and the volume of hippocampus (Xiao et al., 2017; Nilsson et al., 2021). Reduced CSF NPTX-2 level can predict early AD in adults with Down syndrome (Belbin et al., 2020). A longitudinal CSF proteomics study found that the change rate of NPTX-2 concentration was significantly associated with cognitive decline. CSF NPTX-2 was suggested as a strong biomarker for accelerated cognitive decline (Libiger et al., 2021). NPTX-2 regulates the complement activity and the loss of microglial synapses in the brain. Reduced NPTX-2 level may exacerbate complement-mediated neurodegeneration in FTD (Zhou J. et al., 2023). Studies reported significantly reduced CSF NPTX-2 level in FTD (Das et al., 2023) and DLB (Boiten et al., 2020), indicating that CSF NPTX-2 was not a specific AD biomarker. NPTX-2/tau was closely related to the cognition of AD and MCI, and it had the best discrimination on AD (Galasko et al., 2019). The correlation between serum NPTX-2 and AD still remains unclear. There was an independent correlation between the serum NPTX-2 and cognition of VaD patients (Shao et al., 2020). Reduced NPTX-2 level was observed in the plasma NDEs of AD patients, but this change could not be detected 6–11 years before dementia (Goetzl et al., 2018).

α-syn induced hyperphosphorylation and aggregation of tau protein (Duka et al., 2006). There was a correlation between CSF α-syn and MMSE score (Korff et al., 2013). Compared to the cognitively normal population, the level of CSF α-syn in AD increased (Hall et al., 2012). CSF α-syn in AD was significantly higher than those in PD and other neurodegenerative diseases (Wang et al., 2015). More research found that α-syn was a protein associated with familial PD, and it has identified as a major protein of the neuropathological hallmark for idiopathic PD (Fayyad et al., 2019).

### 5.2 Postsynaptic protein

Neurogranin (NG) is the most well-studied postsynaptic protein. It is a protein composed of 78 amino acids, which is involved in synaptic dysfunction or neuronal injury (Represa et al., 1990). NG participates in the induction of synaptic plasticity by accelerating the dissociation of calmodulin, e.g., long-term potentiation (LTP) and long-term depression (LTD) (Kubota et al., 2007). High levels of CSF NG reflected the loss of brain neurogranin protein (Saunders et al., 2023), and it was positively correlated with Aβ plaque and tau pathology (Pereira et al., 2017; Portelius et al., 2018). The content of CSF NG48-76 increased significantly indicating the neurodegenerative process in the brain (Kvartsberg et al., 2015). CSF NG in MCI and AD was higher than cognitive normal population, and it could help to predict the progression from MCI to AD (Kester et al., 2015). CSF NG can predict MCI 5–7 years in advance (Jia et al., 2021), but it could not differentiate MCI from FTD or DLB (Mavroudis et al., 2020). The specificity of CSF NG as a biomarker for AD diagnosis is controversial. Studies indicated that the elevation of CSF NG was highly specific to AD (Portelius et al., 2018), while others presented opposite conclusion (Willemse et al., 2021). Blood NG did not show a good trend. Plasma NG had no significant change or in a similar range between AD and healthy controls (De Vos et al., 2015; Kvartsberg et al., 2015). NG level in NDEVs of MCI and AD decreased, and it was correlated with the cognitive decline (Goetzl et al., 2016; Winston et al., 2016; Liu W. et al., 2020). A meta-analysis reported decreased NG level in plasma exosomes, and plasma NG was closely correlated with the cognitive decline (Liu W. et al., 2020). The interpretation of NDEVs biomarkers should be taken with caution because of their controversial origin (Mahaman et al., 2022).

## 6 Biomarkers of neuroinflammation

Neuropathologist Alois Alzheimer firstly observed lipid accumulation and fibrous structures in glial cells of dementia population’s brain (Simrén et al., 2023). Glial cells in CNS include astrocytes and microglia, and they were reported to be involved in AD pathophysiology (Table 4; Simrén et al., 2023).

**Table 4:** Summarized information of AD neuroinflammation biofluid biomarkers presented in this review.

Pathophysiological mechanism	Biomarker	Biological matrices	Trend	Purpose
Neuroinflammation	TREM2 & sTREM2	CSF	Controversial (Ma et al., 2020)	Research (Hu et al., 2014; Suárez-Calvet et al., 2016b)
		Blood	Increase (Hu et al., 2014)	
	TAM receptor	CSF	Increase (Brosseron et al., 2022, 2023)	Research (Brosseron et al., 2022, 2023)
		Blood		
	YKL-40	CSF	Increase (Janelidze et al., 2016a)	Research (Janelidze et al., 2016a; Villar-Piqué et al., 2019)
		Blood	Increase (Craig-Schapiro et al., 2010)	
	GFAP	CSF	Increase (Benedet et al., 2021)	Research (Benedet et al., 2021; Cicognola et al., 2021; Katsipis et al., 2021)
		Blood	Increase (Benedet et al., 2021)	
		Saliva	Decrease (Katsipis et al., 2021)	
	NRG-1	CSF	Increase (Chang et al., 2016; Mouton-Liger et al., 2020)	Research (Chang et al., 2016; Mouton-Liger et al., 2020)
		Blood		
	TRAIL	Blood	Decrease (Wu et al., 2015)	Research (Wu et al., 2015)

### 6.1 TREM2 and sTREM2

Triggering receptor 2 (TREM2) is a cell surface receptor mainly expressed in CNS microglia, and it plays an important role in regulating energy metabolism and phenotypic transformation of microglia. TREM2 acts as a key regulator, allowing microglia to switch between steady state and activated state (disease-associated microglia) (Keren-Shaul et al., 2017). Heterozygous loss-of-function variants in TREM2 were associated with the increasing AD risk (Guerreiro et al., 2013), and another study presented that they also increased the risk of the other neurodegenerative diseases, such as FTD, PD, and ALS (Carmona et al., 2018). Studies reported that TREM2 tripled the risk of AD (Guerreiro et al., 2013), probably through its influence on tau pathology (Lill et al., 2015) and Aβ clearance pathway (Kleinberger et al., 2014). CSF TREM2 level was deranged at the early stage of AD, and it was closely related to neurodegenerative biomarkers such as T-tau and/or P-tau (Heslegrave et al., 2016; Suárez-Calvet et al., 2016b). Compared to the healthy controls, the levels of TREM2 mRNA and protein in peripheral blood of AD were higher (Hu et al., 2014). TREM2 level in MCI-AD patients was significantly higher than that in AD or healthy controls (Casati et al., 2018).

After hydrolysis of the TREM2 protein, the soluble fragments of sTREM2 are produced outside the cell (Colonna and Wang, 2016). Transgelin-2 (TG2) is expressed on neurons. The sTREM2-TG2 interaction mediates the crosstalk between neurons and microglia (Zhang et al., 2023). The concentration of CSF sTREM2 in AD was higher than the healthy controls (Piccio et al., 2016; Suárez-Calvet et al., 2016b). Before the appearance of expected symptoms and after the increasing of Aβ and T-tau, the level of CSF sTREM2 in ADAD mutation carriers was higher than non-carriers (Suárez-Calvet et al., 2016a). It was suggested that sTREM2 was potential to predict the progression from MCI to AD. The MCI patients with low CSF or high plasma sTREM2 levels had a higher risk of progression to AD. In addition, the concentration of CSF sTREM2 was closely correlated with sTREM2 in plasma (Zhao et al., 2022). The dynamically changes of CSF sTREM2 was observed in preclinical AD. In the absence of neurodegeneration and tau deposition, Aβ pathology was correlated with the decreased CSF sTREM2. However, tau pathology and neurodegeneration are associated with the increased CSF sTREM2 (Ma et al., 2020). A meta-analysis reported that CSF sTREM2 was significantly elevated in the entire continuum of AD compared to controls. However, an increasing trend of CSF sTREM2 was also observed in PD, multiple sclerosis (MS), FTD and DLB (Zhou W. et al., 2023).

### 6.2 TAM receptor

TAM receptors are widely expressed in various types of cells and tissues in the immune, neurological, vascular, and reproductive systems (Zhou S. et al., 2023). Axl and Mertk are expressed in microglia, but Tyro3 is not (Fourgeaud et al., 2016). The TAM system is an important regulator for microglia to recognize and engulfment of amyloid plaques. Axl and Mertk proteins were correlated with AD (Huang et al., 2021). Axl reduces inflammatory responses, while Mertk plays a role in the phagocytosis of aggregated proteins and cellular debris (Zhou S. et al., 2023). Research reported that Aβ plaque-associated microglia exhibited hyperreactivity upon systemic inflammation and upregulated phagocytic genes in the transgenic AD mouse model, including *Axl* (Yin et al., 2017). Microglial phagocytosis driven by TAM does not inhibit, but promotes the formation of amyloid plaques. Mertk is the major participant in this process (Huang et al., 2021). Phosphatidylserine (PtdSer) serves as the ligand decorating the plaques for TAM receptors. Mertk, expressed by activated microglia, recognized, and phagocytosed neurons exposed to PtdSer, thereby promoting tau-induced neuronal loss (Pampuscenko et al., 2020; Zhou S. et al., 2023). The increasing of soluble Axl level in CSF reflected the pathophysiology of AD and was positively correlated with P-tau181 (Brosseron et al., 2022). However, extensive clinical data showed that CSF Axl was correlated with larger brain volume and slower cognitive decline, which indicated that Axl had a protective effect on related processes (Brosseron et al., 2022). The serum Axl was significantly elevated in AD, and it was negatively correlated with cognition and structural imaging (Brosseron et al., 2023). However, the specificity of Axl as an AD biomarker is still required further investigation.

### 6.3 YKL-40

Human cartilage glycoprotein-39 (YKL-40) is a biomarker of inflammation, as well as the activated astrocytes (Olsson et al., 2016). It is primarily produced by astrocytes in the brain and it can predict the neurotoxicity induced by inflammation and other stress signals (Connolly et al., 2023). The research focus on the role of YKL-40 in AD is limited. It was presented that YKL-40 may promote AD progression via altering amyloid burden and neuroinflammatory processes (Connolly et al., 2023). Another study indicated a correlation between YKL-40 and tau pathology (Baldacci et al., 2017a). YKL-40 showed good performance in distinguishing tau-positive patients from controls (Baldacci et al., 2017b). A meta-analysis found that CSF YKL-40 could serve as a biomarker to predict the progression from MCI to AD, as well as for the prognosis of MCI. CSF YKL-40 was significantly elevated in AD, MCI, MCI-AD, and stable MCI compared to the controls, and it was higher in MCI-AD than in the stable MCI (Mavroudis I. et al., 2021). The level of CSF YKL-40 increased in AD and FTD, and it was relatively low in DLB (Craig-Schapiro et al., 2010; Janelidze et al., 2016a). Another study showed that the level of CSF YKL-40 in AD increased compared to non-dementia control groups, DLB and PD (Wennström et al., 2015). The above results suggested that CSF YKL-40 could be used as a non-specific neuroinflammatory biomarker to distinguish AD from PD and DLB (Wennström et al., 2015). The application of plasma YKL-40 in diagnosis of AD is controversial. It was reported that higher plasma YKL-40 in Clinical Dementia Rating (CDR) 1 and 0.5 than CDR 0 (Craig-Schapiro et al., 2010). Another study indicated the possibility of YKL-40 for predicting the progression from MCI to mild AD (Choi et al., 2011). Some studies presented no statistical difference of plasma YKL-40 between AD and controls. However, the significantly increased plasma YKL-40 was observed in CJD and LBD (Craig-Schapiro et al., 2010; Villar-Piqué et al., 2019).

### 6.4 GFAP

Glial fibrillary acidic protein (GFAP) is the main intermediate filament of the glial cytoskeleton (Pekny and Nilsson, 2005). GFAP mediates insulin-like growth factor 1 signaling pathway that involved in AD pathology (Wang et al., 2023). CSF GFAP increased with plaque deposition (Benedet et al., 2021), and it could be used to predict the progression from MCI to AD (Cicognola et al., 2021). In addition, studies found that CSF GFAP can effectively differentiate healthy population from preclinical ADs (Wojdała et al., 2023). Plasma GFAP is considered as an early biomarker for Aβ pathology, but it is not related with tau pathology (Pereira et al., 2021). Plasma GFAP was sensitive to AD pathology in LBD, especially to Aβ plaque accumulation (Cousins et al., 2023). Studies showed that plasma GFAP level continued to increase as the disease progression (AD dementia>MCI > cognitively normal elderly with Aβ positive), but CSF GFAP did not reflect the consistent trend (Verberk et al., 2020; Chatterjee et al., 2021). The reason why plasma GFAP performs better than CSF GFAP as AD biomarker is still unclear. This is probably correlated with the direct release of GFAP into the blood by astrocytic end-feet, and/or different biological degradation between these two matrices (Simrén et al., 2022). Additionally, plasma GFAP can serve as a biomarker for adult AD in Down syndrome, and it could be applied in clinical practice and trials (Montoliu-Gaya et al., 2023). Notably, significantly decreasing GFAP concentration was observed in saliva of MCI and AD compared to controls. Salivary GFAP is suggested as an excellent biomarker for distinguishing MCI or AD from the controls (Katsipis et al., 2021).

### 6.5 NRG-1

Neuregulin (NRG) plays an important role in the development of nervous system (Esper et al., 2006). NRG-1 is the first and best characterized NRG gene (Pankonin et al., 2009). Soluble NRG (sNRG) was preferentially accumulated on the surface of white matter astrocytes (Pankonin et al., 2009). NRG-1 signaling exerts neuroprotection through the activation of the phosphatidylinositol 3-kinase/Akt (PI3K/Akt) pathway to prevent the neurotoxicity induced by Aβ_42_ (Baik et al., 2016). Another study presented prevention of AD via blocking NRG-1 signaling on microglia (Liu et al., 2023). Both the above studies are based on the animal models. Research found that ADs and MCI-ADs had higher CSF NRG-1 than controls and non-AD dementias, and the CSF NRG-1 was correlated with cognitive evolution (Mouton-Liger et al., 2020). Plasma sNRG-1 level in AD increased and there was a significant correlation between plasma sNRG-1 and MMSE score. The lower MMSE score was correlated with the higher plasma sNRG-1 concentration (Chang et al., 2016). In addition, plasma NRG-1 was found to be correlated with CSF GAP-43, SNAP-25, and NG (Vrillon et al., 2022). Therefore, sNRG-1 was suggested as a sensitive plasma biomarker (Chang et al., 2016).

### 6.6 TRAIL

Previous study presented the involvement of TNF-related apoptosis-inducing ligand (TRAIL) in Aβ induced neurotoxicity in a human neuronal cell line (Cantarella et al., 2003). Blockade of the TRAIL-death receptor DR5 prevented Aβ-neurotoxicity (Uberti et al., 2007). It was reported that the TRAIL was specifically expressed in the brains of ADs (Uberti et al., 2004). The reduced plasma TRAIL level was observed in AD group compared to controls (Wu et al., 2015), and it was significantly associated with its level in CSF (Wu et al., 2015). No significant difference was observed in the serum TRAIL between ADs and controls. However, a negative correlation was indicated between serum TRAIL and MMSE scores of AD patients (Genc et al., 2009).

## 7 Biomarkers of BBB breakdown

*ApoE* ε4 could accelerate the breakdown of BBB and damage the pericapillary cells of the brain (Halliday et al., 2016). High level of platelet-derived growth factor receptor-β (PDGFR-β) in CSF could predict the cognitive decline of *ApoE* ε4 carriers (Montagne et al., 2020). Brain capillary injury and BBB breakdown occurred in the hippocampus of patients with early cognitive impairment (Nation et al., 2019). However, the BBB disruptions were not correlated with alterations in Aβ and/or tau levels (Nation et al., 2019; Montagne et al., 2020). BBB breakdown was proposed as an early biomarker for human cognitive impairment that was uncorrelated to Aβ and tau pathology (Mahaman et al., 2022).

The ratio of CSF to serum albumin was applied as a standard method to measure BBB function (Tibbling et al., 1977; Skillbäck et al., 2017; Menendez-Gonzalez and Gasparovic, 2019). The mean value of this ratio was slightly higher in AD patients with vascular factors when compared to the healthy controls, but no significant difference was observed in AD group without vascular factors (Blennow et al., 1990). It is suggested that the BBB damage in AD was associated with clinical vascular factors but not a result of the disease itself (Blennow et al., 1990). This ratio might help to exclude some of the cerebrovascular diseases and indirectly assist the diagnosis of AD (Reitz and Mayeux, 2014).

## 8 Outlook

The Ronald and Nancy Reagan Research Institute of the Alzheimer’s Association, and the National Institute on Aging Working Group proposed that “ideal” biomarkers for AD diagnosis should meet the following criteria (Khan and Alkon, 2015): (1) the basic characteristics of AD neuropathology could be detected; (2) the application of biomarkers should be validated in neuropathologically confirmed AD cases; (3) biomarkers could diagnose AD at the early stage with high sensitivity and specificity; and (4) the analysis should be repeatable, reliable, non-invasive, simple and cost-effective (Kulichikhin et al., 2021). Several challenges still exist. First, the diagnosis of AD mainly depends on the clinical features. Some clinical features are overlapped between AD and non-AD dementia patients, resulting in a high rate of misdiagnosis at the early stage of AD, especially in non-specialist clinical centers (Engelborghs et al., 2008). Second, other dementias, e.g., VaD and DLB, have the pathological characteristics of AD (Schneider et al., 2009), which hinders the application of some AD biomarkers. Third, Aβ plaques and NFT are also presented in the elderly population without cognitive impairment (Price and Morris, 1999). Fourth, the detection of most AD biomarkers has not been standardized. Most of the results are preliminary and retrospective, and lacking comparison between patients (Sunderland et al., 2003; Mahaman et al., 2022). Finally, more meta-analysis or the correlation analysis studies among different biomarkers are insufficient. Further investigation to rank and confirm their importance is required.

In the future, the research could focus on demonstrating the accuracy of AD biomarkers, especially for the blood-based biomarkers. The relationship between AD biomarkers and the pathogenesis of AD needs further exploration. Large-scale head-to-head studies are required to determine the most appropriate application scenario of biomarkers at different stages of AD, involving diagnosis, prediction, prognosis, and clinical trial design (Ossenkoppele et al., 2019).

## Author contributions

SW: Writing – review & editing, Writing – original draft. SX: Writing – review & editing, Writing – original draft. QZ: Writing – original draft. ZZ: Writing – original draft. TW: Writing – review & editing, Supervision, Conceptualization. GZ: Writing – review & editing, Supervision, Conceptualization.

## References

[ref1] Agliardi C. Guerini F. R. Zanzottera M. Bianchi A. Nemni R. Clerici M. (2019). SNAP-25 in serum is carried by exosomes of neuronal origin and is a potential biomarker of Alzheimer’s disease. Mol. Neurobiol. 56, 5792–5798. doi: 10.1007/s12035-019-1501-x 30680692

[ref2] Alzheimer’s Disease International (2019). Alzheimer’s disease international: world Alzheimer report 2019. London: Alzheimer’s Disease International.

[ref3] Ashton N. J. Pascoal T. A. Karikari T. K. Benedet A. L. Lantero-Rodriguez J. Brinkmalm G.. (2021). Plasma p-tau231: a new biomarker for incipient Alzheimer's disease pathology. Acta Neuropathol. 141, 709–724. doi: 10.1007/s00401-021-02275-6 33585983 PMC8043944

[ref4] Attems J. Jellinger K. Thal D. R. Van Nostrand W. (2011). Review: sporadic cerebral amyloid angiopathy. Neuropathol. Appl. Neurobiol. 37, 75–93. doi: 10.1111/j.1365-2990.2010.01137.x 20946241

[ref5] Baik T. K. Kim Y. J. Kang S. M. Song D. Y. Min S. S. Woo R. S. (2016). Blocking the phosphatidylinositol 3-kinase pathway inhibits neuregulin-1-mediated rescue of neurotoxicity induced by Aβ1-42. J. Pharm. Pharmacol. 68, 1021–1029. doi: 10.1111/jphp.12563 27230708

[ref6] Baldacci F. Lista S. Cavedo E. Bonuccelli U. Hampel H. (2017a). Diagnostic function of the neuroinflammatory biomarker YKL-40 in Alzheimer's disease and other neurodegenerative diseases. Expert Rev. Proteomics 14, 285–299. doi: 10.1080/14789450.2017.1304217 28281838

[ref7] Baldacci F. Toschi N. Lista S. Zetterberg H. Blennow K. Kilimann I.. (2017b). Two-level diagnostic classification using cerebrospinal fluid YKL-40 in Alzheimer's disease. Alzheimers Dement. 13, 993–1003. doi: 10.1016/j.jalz.2017.01.021 28263742

[ref8] Baldeiras I. Santana I. Leitão M. J. Gens H. Pascoal R. Tábuas-Pereira M.. (2018). Addition of the Aβ42/40 ratio to the cerebrospinal fluid biomarker profile increases the predictive value for underlying Alzheimer’s disease dementia in mild cognitive impairment. Alzheimers Res. Ther. 10:33. doi: 10.1186/s13195-018-0362-2 29558986 PMC5861634

[ref9] Barthélemy N. R. Horie K. Sato C. Bateman R. J. (2020a). Blood plasma phosphorylated-tau isoforms track CNS change in Alzheimer’s disease. J. Exp. Med. 217:e20200861. doi: 10.1084/jem.20200861 32725127 PMC7596823

[ref10] Barthélemy N. R. Li Y. Joseph-Mathurin N. Gordon B. A. Hassenstab J. Benzinger T. L. S.. (2020b). A soluble phosphorylated tau signature links tau, amyloid and the evolution of stages of dominantly inherited Alzheimer’s disease. Nat. Med. 26, 398–407. doi: 10.1038/s41591-020-0781-z 32161412 PMC7309367

[ref11] Barthélemy N. R. Mallipeddi N. Moiseyev P. Sato C. Bateman R. J. (2019). Tau Phosphorylation Rates Measured by Mass Spectrometry Differ in the Intracellular Brain vs. Extracellular Cerebrospinal Fluid Compartments and Are Differentially Affected by Alzheimer's Disease. Front. Aging Neurosci. 11:121. doi: 10.3389/fnagi.2019.00121 31178717 PMC6537657

[ref12] Bateman R. J. Xiong C. Benzinger T. L. Fagan A. M. Goate A. Fox N. C.. (2012). Clinical and biomarker changes in dominantly inherited Alzheimer's disease. N. Engl. J. Med. 367, 795–804. doi: 10.1056/NEJMoa1202753 22784036 PMC3474597

[ref13] Belbin O. Xiao M. F. Xu D. Carmona-Iragui M. Pegueroles J. Benejam B.. (2020). Cerebrospinal fluid profile of NPTX2 supports role of Alzheimer's disease-related inhibitory circuit dysfunction in adults with Down syndrome. Mol. Neurodegener. 15:46. doi: 10.1186/s13024-020-00398-0 32807227 PMC7433053

[ref14] Benedet A. L. Milà-Alomà M. Vrillon A. Ashton N. J. Pascoal T. A. Lussier F.. (2021). Differences Between Plasma and Cerebrospinal Fluid Glial Fibrillary Acidic Protein Levels Across the Alzheimer Disease Continuum. JAMA Neurol. 78, 1471–1483. doi: 10.1001/jamaneurol.2021.3671 34661615 PMC8524356

[ref15] Blennow K. Chen C. Cicognola C. Wildsmith K. R. Manser P. T. Bohorquez S. M. S.. (2020). Cerebrospinal fluid tau fragment correlates with tau PET: a candidate biomarker for tangle pathology. Brain 143, 650–660. doi: 10.1093/brain/awz346 31834365 PMC7009597

[ref16] Blennow K. De Leon M. J. Zetterberg H. (2006). Alzheimer's disease. Lancet 368, 387–403. doi: 10.1016/s0140-6736(06)69113-7 16876668

[ref17] Blennow K. Hampel H. (2003). CSF markers for incipient Alzheimer's disease. Lancet Neurol. 2, 605–613. doi: 10.1016/s1474-4422(03)00530-1 14505582

[ref18] Blennow K. Wallin A. Fredman P. Karlsson I. Gottfries C. G. Svennerholm L. (1990). Blood-brain barrier disturbance in patients with Alzheimer's disease is related to vascular factors. Acta Neurol. Scand. 81, 323–326. doi: 10.1111/j.1600-0404.1990.tb01563.x 2360400

[ref19] Blennow K. Zetterberg H. (2018). Biomarkers for Alzheimer's disease: current status and prospects for the future. J. Intern. Med. 284, 643–663. doi: 10.1111/joim.12816 30051512

[ref20] Boiten W. A. Van Steenoven I. Xiao M. F. Worley P. F. Noli B. Cocco C.. (2020). Pathologically Decreased CSF Levels of Synaptic Marker NPTX2 in DLB Are Correlated with Levels of Alpha-Synuclein and VGF. Cells 10:38. doi: 10.3390/cells10010038 33383752 PMC7824459

[ref21] Braunewell K. H. (2012). The visinin-like proteins VILIP-1 and VILIP-3 in Alzheimer's disease-old wine in new bottles. Front. Mol. Neurosci. 5:20. doi: 10.3389/fnmol.2012.00020 22375104 PMC3284765

[ref22] Braunewell K. Riederer P. Spilker C. Gundelfinger E. D. Bogerts B. Bernstein H. G. (2001). Abnormal localization of two neuronal calcium sensor proteins, visinin-like proteins (vilips)-1 and −3, in neocortical brain areas of Alzheimer disease patients. Dement. Geriatr. Cogn. Disord. 12, 110–116. doi: 10.1159/000051244 11173883

[ref23] Bridel C. Van Wieringen W. N. Zetterberg H. Tijms B. M. Teunissen C. E. Alvarez-Cermeño J. C.. (2019). Diagnostic Value of Cerebrospinal Fluid Neurofilament Light Protein in Neurology: A Systematic Review and Meta-analysis. JAMA Neurol. 76, 1035–1048. doi: 10.1001/jamaneurol.2019.1534 31206160 PMC6580449

[ref24] Brosseron F. Maass A. Kleineidam L. Ravichandran K. A. González P. G. Mcmanus R. M.. (2022). Soluble TAM receptors sAXL and sTyro3 predict structural and functional protection in Alzheimer's disease. Neuron 110, 1009–1022.e4. doi: 10.1016/j.neuron.2021.12.016 34995486

[ref25] Brosseron F. Maass A. Kleineidam L. Ravichandran K. A. Kolbe C. C. Wolfsgruber S.. (2023). Serum IL-6, sAXL, and YKL-40 as systemic correlates of reduced brain structure and function in Alzheimer's disease: results from the DELCODE study. Alzheimers Res. Ther. 15:13. doi: 10.1186/s13195-022-01118-0 36631909 PMC9835320

[ref26] Bruno D. Pomara N. Nierenberg J. Ritchie J. C. Lutz M. W. Zetterberg H.. (2012). Levels of cerebrospinal fluid neurofilament light protein in healthy elderly vary as a function of TOMM40 variants. Exp. Gerontol. 47, 347–352. doi: 10.1016/j.exger.2011.09.008 21983493 PMC4550703

[ref27] Buchhave P. Minthon L. Zetterberg H. Wallin A. K. Blennow K. Hansson O. (2012). Cerebrospinal fluid levels of β-amyloid 1-42, but not of tau, are fully changed already 5 to 10 years before the onset of Alzheimer dementia. Arch. Gen. Psychiatry 69, 98–106. doi: 10.1001/archgenpsychiatry.2011.155 22213792

[ref28] Burgoyne R. D. Weiss J. L. (2001). The neuronal calcium sensor family of Ca2+−binding proteins. Biochem. J. 353, 1–12. 11115393 PMC1221537

[ref29] Cantarella G. Uberti D. Carsana T. Lombardo G. Bernardini R. Memo M. (2003). Neutralization of TRAIL death pathway protects human neuronal cell line from beta-amyloid toxicity. Cell Death Differ. 10, 134–141. doi: 10.1038/sj.cdd.4401143 12655302

[ref30] Carmona S. Zahs K. Wu E. Dakin K. Bras J. Guerreiro R. (2018). The role of TREM2 in Alzheimer's disease and other neurodegenerative disorders. Lancet Neurol. 17, 721–730. doi: 10.1016/s1474-4422(18)30232-1 30033062

[ref31] Casati M. Ferri E. Gussago C. Mazzola P. Abbate C. Bellelli G.. (2018). Increased expression of TREM2 in peripheral cells from mild cognitive impairment patients who progress into Alzheimer's disease. Eur. J. Neurol. 25, 805–810. doi: 10.1111/ene.13583 29377401

[ref32] Cervellati C. Trentini A. Rosta V. Passaro A. Bosi C. Sanz J. M.. (2020). Serum beta-secretase 1 (BACE1) activity as candidate biomarker for late-onset Alzheimer's disease. Geroscience 42, 159–167. doi: 10.1007/s11357-019-00127-6 31745860 PMC7031490

[ref33] Chang K. A. Shin K. Y. Nam E. Lee Y. B. Moon C. Suh Y. H.. (2016). Plasma soluble neuregulin-1 as a diagnostic biomarker for Alzheimer's disease. Neurochem. Int. 97, 1–7. doi: 10.1016/j.neuint.2016.04.012 27133777

[ref34] Chatterjee P. Pedrini S. Stoops E. Goozee K. Villemagne V. L. Asih P. R.. (2021). Plasma glial fibrillary acidic protein is elevated in cognitively normal older adults at risk of Alzheimer's disease. Transl. Psychiatry 11:27. doi: 10.1038/s41398-020-01137-1 33431793 PMC7801513

[ref35] Chen T. B. Lee Y. J. Lin S. Y. Chen J. P. Hu C. J. Wang P. N.. (2019). Plasma Aβ42 and Total Tau Predict Cognitive Decline in Amnestic Mild Cognitive Impairment. Sci. Rep. 9:13984. doi: 10.1038/s41598-019-50315-9 31562355 PMC6764975

[ref36] Choi J. Lee H. W. Suk K. (2011). Plasma level of chitinase 3-like 1 protein increases in patients with early Alzheimer's disease. J. Neurol. 258, 2181–2185. doi: 10.1007/s00415-011-6087-9 21562723

[ref37] Cicognola C. Janelidze S. Hertze J. Zetterberg H. Blennow K. Mattsson-Carlgren N.. (2021). Plasma glial fibrillary acidic protein detects Alzheimer pathology and predicts future conversion to Alzheimer dementia in patients with mild cognitive impairment. Alzheimers Res. Ther. 13:68. doi: 10.1186/s13195-021-00804-9 33773595 PMC8005231

[ref38] Colonna M. Wang Y. (2016). TREM2 variants: new keys to decipher Alzheimer disease pathogenesis. Nat. Rev. Neurosci. 17, 201–207. doi: 10.1038/nrn.2016.7 26911435

[ref39] Connolly K. Lehoux M. O'rourke R. Assetta B. Erdemir G. A. Elias J. A.. (2023). Potential role of chitinase-3-like protein 1 (CHI3L1/YKL-40) in neurodegeneration and Alzheimer's disease. Alzheimers Dement. 19, 9–24. doi: 10.1002/alz.12612 35234337 PMC9437141

[ref40] Cousins K. Irwin D. J. Chen-Plotkin A. Shaw L. M. Arezoumandan S. Lee E. B.. (2023). Plasma GFAP associates with secondary Alzheimer's pathology in Lewy body disease. Ann. Clin. Transl. Neurol. 10, 802–813. doi: 10.1002/acn3.51768, PMID: 37000892 PMC10187730

[ref41] Craig-Schapiro R. Perrin R. J. Roe C. M. Xiong C. Carter D. Cairns N. J.. (2010). YKL-40: a novel prognostic fluid biomarker for preclinical Alzheimer's disease. Biol. Psychiatry 68, 903–912. doi: 10.1016/j.biopsych.2010.08.025 21035623 PMC3011944

[ref42] Crotti A. Sait H. R. Mcavoy K. M. Estrada K. Ergun A. Szak S.. (2019). BIN1 favors the spreading of Tau via extracellular vesicles. Sci. Rep. 9:9477. doi: 10.1038/s41598-019-45676-0 31263146 PMC6603165

[ref43] Das S. Goossens J. Jacobs D. Dewit N. Pijnenburg Y. In ‘t Veld S. G. J. G.. (2023). Synaptic biomarkers in the cerebrospinal fluid associate differentially with classical neuronal biomarkers in patients with Alzheimer’s disease and frontotemporal dementia. Alzheimers Res. Ther. 15:62. doi: 10.1186/s13195-023-01212-x, PMID: 36964594 PMC10037899

[ref44] Davidsson P. Blennow K. (1998). Neurochemical dissection of synaptic pathology in Alzheimer’s disease. Int. Psychogeriatr. 10, 11–23. doi: 10.1017/s1041610298005110 9629521

[ref45] De Jong D. Jansen R. W. Pijnenburg Y. A. Van Geel W. J. Borm G. F. Kremer H. P.. (2007). CSF neurofilament proteins in the differential diagnosis of dementia. J. Neurol. Neurosurg. Psychiatry 78, 936–938. doi: 10.1136/jnnp.2006.107326 17314187 PMC2117885

[ref46] De Vos A. Jacobs D. Struyfs H. Fransen E. Andersson K. Portelius E.. (2015). C-terminal neurogranin is increased in cerebrospinal fluid but unchanged in plasma in Alzheimer's disease. Alzheimers Dement. 11, 1461–1469. doi: 10.1016/j.jalz.2015.05.012 26092348

[ref47] Decourt B. Sabbagh M. N. (2011). BACE1 as a potential biomarker for Alzheimer's disease. J. Alzheimers Dis. 24, 53–59. doi: 10.3233/jad-2011-110017 21403391 PMC3313825

[ref48] Duka T. Rusnak M. Drolet R. E. Duka V. Wersinger C. Goudreau J. L.. (2006). Alpha-synuclein induces hyperphosphorylation of Tau in the MPTP model of parkinsonism. FASEB J. 20, 2302–2312. doi: 10.1096/fj.06-6092com 17077307

[ref49] Engelborghs S. De Vreese K. Van De Casteele T. Vanderstichele H. Van Everbroeck B. Cras P.. (2008). Diagnostic performance of a CSF-biomarker panel in autopsy-confirmed dementia. Neurobiol. Aging 29, 1143–1159. doi: 10.1016/j.neurobiolaging.2007.02.016 17428581

[ref50] Esper R. M. Pankonin M. S. Loeb J. A. (2006). Neuregulins: versatile growth and differentiation factors in nervous system development and human disease. Brain Res. Rev. 51, 161–175. doi: 10.1016/j.brainresrev.2005.11.006 16412517

[ref51] Fagan A. M. Mintun M. A. Mach R. H. Lee S. Y. Dence C. S. Shah A. R.. (2006). Inverse relation between in vivo amyloid imaging load and cerebrospinal fluid Abeta42 in humans. Ann. Neurol. 59, 512–519. doi: 10.1002/ana.20730 16372280

[ref52] Fayyad M. Salim S. Majbour N. Erskine D. Stoops E. Mollenhauer B.. (2019). Parkinson's disease biomarkers based on α-synuclein. J. Neurochem. 150, 626–636. doi: 10.1111/jnc.14809 31265130

[ref53] Fleisher A. S. Chen K. Quiroz Y. T. Jakimovich L. J. Gutierrez Gomez M. Langois C. M.. (2015). Associations between biomarkers and age in the presenilin 1 E280A autosomal dominant Alzheimer disease kindred: a cross-sectional study. JAMA Neurol. 72, 316–324. doi: 10.1001/jamaneurol.2014.3314 25580592 PMC4355261

[ref54] Fortea J. Carmona-Iragui M. Benejam B. Fernández S. Videla L. Barroeta I.. (2018). Plasma and CSF biomarkers for the diagnosis of Alzheimer's disease in adults with Down syndrome: a cross-sectional study. Lancet Neurol. 17, 860–869. doi: 10.1016/s1474-4422(18)30285-0 30172624

[ref55] Fourgeaud L. Través P. G. Tufail Y. Leal-Bailey H. Lew E. D. Burrola P. G.. (2016). TAM receptors regulate multiple features of microglial physiology. Nature 532, 240–244. doi: 10.1038/nature17630 27049947 PMC5358512

[ref56] Frisoni G. B. Fox N. C. Jack C. R. Jr. Scheltens P. Thompson P. M. (2010). The clinical use of structural MRI in Alzheimer disease. Nat. Rev. Neurol. 6, 67–77. doi: 10.1038/nrneurol.2009.215 20139996 PMC2938772

[ref57] Galasko D. Xiao M. Xu D. Smirnov D. Salmon D. P. Dewit N.. (2019). Synaptic biomarkers in CSF aid in diagnosis, correlate with cognition and predict progression in MCI and Alzheimer's disease. Alzheimers Dement 5, 871–882. doi: 10.1016/j.trci.2019.11.002 PMC6911971 31853477

[ref58] Gauthier S. Wevster C. Servaes S. Morais J. A. Rosa-Neto P. (2022). World Alzheimer report 2022 life after diagnosis: navigating treatment, care and support. (London, England: Alzheimer’s Disease International).

[ref59] Genc S. Egrilmez M. Y. Yaka E. Cavdar Z. Iyilikci L. Yener G.. (2009). TNF-related apoptosis-inducing ligand level in Alzheimer's disease. Neurol. Sci. 30, 263–267. doi: 10.1007/s10072-009-0047-5 19294332

[ref60] Geppert M. Goda Y. Hammer R. E. Li C. Rosahl T. W. Stevens C. F.. (1994). Synaptotagmin I: a major Ca2+ sensor for transmitter release at a central synapse. Cell 79, 717–727. doi: 10.1016/0092-8674(94)90556-8 7954835

[ref61] Goetzl E. J. Abner E. L. Jicha G. A. Kapogiannis D. Schwartz J. B. (2018). Declining levels of functionally specialized synaptic proteins in plasma neuronal exosomes with progression of Alzheimer's disease. FASEB J. 32, 888–893. doi: 10.1096/fj.201700731R 29025866 PMC5888398

[ref62] Goetzl E. J. Kapogiannis D. Schwartz J. B. Lobach I. V. Goetzl L. Abner E. L.. (2016). Decreased synaptic proteins in neuronal exosomes of frontotemporal dementia and Alzheimer's disease. FASEB J. 30, 4141–4148. doi: 10.1096/fj.201600816R 27601437 PMC5102122

[ref63] Gong C. X. Lidsky T. Wegiel J. Zuck L. Grundke-Iqbal I. Iqbal K. (2000). Phosphorylation of microtubule-associated protein tau is regulated by protein phosphatase 2A in mammalian brain. Implications for neurofibrillary degeneration in Alzheimer's disease. J. Biol. Chem. 275, 5535–5544. doi: 10.1074/jbc.275.8.5535 10681533

[ref64] Grundke-Iqbal I. Iqbal K. Tung Y. C. Quinlan M. Wisniewski H. M. Binder L. I. (1986). Abnormal phosphorylation of the microtubule-associated protein tau (tau) in Alzheimer cytoskeletal pathology. Proc. Natl. Acad. Sci. U. S. A. 83, 4913–4917. doi: 10.1073/pnas.83.13.4913 3088567 PMC323854

[ref65] Guerreiro R. Wojtas A. Bras J. Carrasquillo M. Rogaeva E. Majounie E.. (2013). TREM2 variants in Alzheimer’s disease. N. Engl. J. Med. 368, 117–127. doi: 10.1056/NEJMoa1211851 23150934 PMC3631573

[ref66] Haas D. (2018). “Chapter 15- Biomarker for Alzheimer’s disease” in Precision medicine. eds. Deigner H.-P. Kohl M. (London: Academic Press)

[ref67] Halbgebauer S. Steinacker P. Hengge S. Oeckl P. Abu Rumeileh S. Anderl-Straub S.. (2022a). CSF levels of SNAP-25 are increased early in Creutzfeldt-Jakob and Alzheimer's disease. J. Neurol. Neurosurg. Psychiatry. doi: 10.1136/jnnp-2021-328646 35995553

[ref68] Halbgebauer S. Steinacker P. Riedel D. Oeckl P. Anderl-Straub S. Lombardi J.. (2022b). Visinin-like protein 1 levels in blood and CSF as emerging markers for Alzheimer's and other neurodegenerative diseases. Alzheimers Res. Ther. 14:175. doi: 10.1186/s13195-022-01122-4 36419075 PMC9682835

[ref69] Hall S. Öhrfelt A. Constantinescu R. Andreasson U. Surova Y. Bostrom F.. (2012). Accuracy of a panel of 5 cerebrospinal fluid biomarkers in the differential diagnosis of patients with dementia and/or parkinsonian disorders. Arch. Neurol. 69, 1445–1452. doi: 10.1001/archneurol.2012.1654 22925882

[ref70] Halliday M. R. Rege S. V. Ma Q. Zhao Z. Miller C. A. Winkler E. A.. (2016). Accelerated pericyte degeneration and blood-brain barrier breakdown in apolipoprotein E4 carriers with Alzheimer's disease. J. Cereb. Blood Flow Metab. 36, 216–227. doi: 10.1038/jcbfm.2015.44 25757756 PMC4758554

[ref71] Hampel H. Buerger K. Zinkowski R. Teipel S. J. Goernitz A. Andreasen N.. (2004). Measurement of Phosphorylated Tau Epitopes in the Differential Diagnosisof Alzheimer Disease: A Comparative Cerebrospinal Fluid Study. Arch. Gen. Psychiatry 61, 95–102. doi: 10.1001/archpsyc.61.1.95 14706948

[ref72] Hampel H. Lista S. Vanmechelen E. Zetterberg H. Giorgi F. S. Galgani A.. (2020). β-Secretase1 biological markers for Alzheimer's disease: state-of-art of validation and qualification. Alzheimers Res. Ther. 12:130. doi: 10.1186/s13195-020-00686-3 33066807 PMC7566058

[ref73] Hampel H. Vassar R. de Strooper B. Hardy J. Willem M. Singh N.. (2021). The β-Secretase BACE1 in Alzheimer’s Disease. Biol. Psychiatry 89, 745–756. doi: 10.1016/j.biopsych.2020.02.001, PMID: 32223911 PMC7533042

[ref74] Hansson O. Janelidze S. Hall S. Magdalinou N. Lees A. J. Andreasson U.. (2017). Blood-based NfL: A biomarker for differential diagnosis of parkinsonian disorder. Neurology 88, 930–937. doi: 10.1212/wnl.0000000000003680 28179466 PMC5333515

[ref75] Hansson O. Lehmann S. Otto M. Zetterberg H. Lewczuk P. (2019). Advantages and disadvantages of the use of the CSF Amyloid β (Aβ) 42/40 ratio in the diagnosis of Alzheimer’s Disease. Alzheimers Res. Ther. 11:34. doi: 10.1186/s13195-019-0485-0 31010420 PMC6477717

[ref76] Hansson O. Zetterberg H. Buchhave P. Londos E. Blennow K. Minthon L. (2006). Association between CSF biomarkers and incipient Alzheimer's disease in patients with mild cognitive impairment: a follow-up study. Lancet Neurol. 5, 228–234. doi: 10.1016/s1474-4422(06)70355-6 16488378

[ref77] Heslegrave A. Heywood W. Paterson R. Magdalinou N. Svensson J. Johansson P.. (2016). Increased cerebrospinal fluid soluble TREM2 concentration in Alzheimer's disease. Mol. Neurodegener. 11:3. doi: 10.1186/s13024-016-0071-x 26754172 PMC4709982

[ref78] Holmberg B. Johnels B. Blennow K. Rosengren L. (2003). Cerebrospinal fluid Abeta42 is reduced in multiple system atrophy but normal in Parkinson's disease and progressive supranuclear palsy. Mov. Disord. 18, 186–190. doi: 10.1002/mds.10321 12539213

[ref79] Horie K. Barthélemy N. R. Sato C. Bateman R. J. (2021). CSF tau microtubule binding region identifies tau tangle and clinical stages of Alzheimer's disease. Brain 144, 515–527. doi: 10.1093/brain/awaa373 33283854 PMC7940175

[ref80] Hu N. Tan M. S. Yu J. T. Sun L. Tan L. Wang Y. L.. (2014). Increased expression of TREM2 in peripheral blood of Alzheimer's disease patients. J. Alzheimers Dis. 38, 497–501. doi: 10.3233/jad-130854 24002183

[ref81] Huang Y. Happonen K. E. Burrola P. G. O'connor C. Hah N. Huang L.. (2021). Microglia use TAM receptors to detect and engulf amyloid β plaques. Nat. Immunol. 22, 586–594. doi: 10.1038/s41590-021-00913-5 33859405 PMC8102389

[ref82] Jack C. R. Jr. Bennett D. A. Blennow K. Carrillo M. C. Dunn B. Haeberlein S. B.. (2018). NIA-AA Research Framework: Toward a biological definition of Alzheimer's disease. Alzheimers Dement. 14, 535–562. doi: 10.1016/j.jalz.2018.02.018 29653606 PMC5958625

[ref83] Jackson J. Jambrina E. Li J. Marston H. Menzies F. Phillips K.. (2019). Targeting the Synapse in Alzheimer's Disease. Front. Neurosci. 13:735. doi: 10.3389/fnins.2019.00735 31396031 PMC6664030

[ref84] Jan A. Gokce O. Luthi-Carter R. Lashuel H. A. (2008). The ratio of monomeric to aggregated forms of Abeta40 and Abeta42 is an important determinant of amyloid-beta aggregation, fibrillogenesis, and toxicity. J. Biol. Chem. 283, 28176–28189. doi: 10.1074/jbc.M803159200 18694930 PMC2661389

[ref85] Janelidze S. Hertze J. Zetterberg H. Landqvist Waldö M. Santillo A. Blennow K.. (2016a). Cerebrospinal fluid neurogranin and YKL-40 as biomarkers of Alzheimer’s disease. Ann. Clin. Transl. Neurol. 3, 12–20. doi: 10.1002/acn3.266 26783546 PMC4704480

[ref86] Janelidze S. Mattsson N. Palmqvist S. Smith R. Beach T. G. Serrano G. E.. (2020). Plasma P-tau181 in Alzheimer's disease: relationship to other biomarkers, differential diagnosis, neuropathology and longitudinal progression to Alzheimer's dementia. Nat. Med. 26, 379–386. doi: 10.1038/s41591-020-0755-1 32123385

[ref87] Janelidze S. Stomrud E. Palmqvist S. Zetterberg H. Van Westen D. Jeromin A.. (2016b). Plasma β-amyloid in Alzheimer's disease and vascular disease. Sci. Rep. 6:26801. doi: 10.1038/srep26801 27241045 PMC4886210

[ref88] Janelidze S. Zetterberg H. Mattsson N. Palmqvist S. Vanderstichele H. Lindberg O.. (2016c). CSF Aβ42/Aβ40 and Aβ42/Aβ38 ratios: better diagnostic markers of Alzheimer disease. Ann. Clin. Transl. Neurol. 3, 154–165. doi: 10.1002/acn3.274 27042676 PMC4774260

[ref89] Jia L. Qiu Q. Zhang H. Chu L. Du Y. Zhang J.. (2019). Concordance between the assessment of Aβ42, T-tau, and P-T181-tau in peripheral blood neuronal-derived exosomes and cerebrospinal fluid. Alzheimers Dement. 15, 1071–1080. doi: 10.1016/j.jalz.2019.05.002 31422798

[ref90] Jia J. Zhang Y. Shi Y. Yin X. Wang S. Li Y.. (2022). A 19-Year-Old Adolescent with Probable Alzheimer’s Disease 1. J. Alzheimers Dis. 91, 915–922. doi: 10.3233/JAD-221065 36565128

[ref91] Jia L. Zhu M. Kong C. Pang Y. Zhang H. Qiu Q.. (2021). Blood neuro-exosomal synaptic proteins predict Alzheimer's disease at the asymptomatic stage. Alzheimers Dement. 17, 49–60. doi: 10.1002/alz.12166 32776690 PMC7984076

[ref92] Kaneko N. Nakamura A. Washimi Y. Kato T. Sakurai T. Arahata Y.. (2014). Novel plasma biomarker surrogating cerebral amyloid deposition. Proc. Jpn. Acad. Ser. B Phys. Biol. Sci. 90, 353–364. doi: 10.2183/pjab.90.353 PMC4324927 25391320

[ref93] Kang J. Tian Z. Wei J. Mu Z. Liang J. Li M. (2022). Association between obstructive sleep apnea and Alzheimer's disease-related blood and cerebrospinal fluid biomarkers: a meta-analysis. J. Clin. Neurosci. 102, 87–94. doi: 10.1016/j.jocn.2022.06.004 35753156

[ref94] Kaplow J. Vandijck M. Gray J. Kanekiyo M. Huyck E. Traynham C. J.. (2020). Concordance of Lumipulse cerebrospinal fluid t-tau/Aβ42 ratio with amyloid PET status. Alzheimers Dement. 16, 144–152. doi: 10.1002/alz.12000 31914216 PMC7061432

[ref95] Kapogiannis D. Mustapic M. Shardell M. D. Berkowitz S. T. Diehl T. C. Spangler R. D.. (2019). Association of Extracellular Vesicle Biomarkers With Alzheimer Disease in the Baltimore Longitudinal Study of Aging. JAMA Neurol. 76, 1340–1351. doi: 10.1001/jamaneurol.2019.2462 31305918 PMC6632160

[ref96] Karran E. De Strooper B. (2022). The amyloid hypothesis in Alzheimer disease: new insights from new therapeutics. Nat. Rev. Drug Discov. 21, 306–318. doi: 10.1038/s41573-022-00391-w 35177833

[ref97] Katsipis G. Tzekaki E. E. Tsolaki M. Pantazaki A. A. (2021). Salivary GFAP as a potential biomarker for diagnosis of mild cognitive impairment and Alzheimer's disease and its correlation with neuroinflammation and apoptosis. J. Neuroimmunol. 361:577744. doi: 10.1016/j.jneuroim.2021.577744 34655990

[ref98] Kawahara M. Kuroda Y. (2000). Molecular mechanism of neurodegeneration induced by Alzheimer’s β-amyloid protein: channel formation and disruption of calcium homeostasis. Brain Res. Bull. 53, 389–397. doi: 10.1016/S0361-9230(00)00370-1 11136994

[ref99] Keren-Shaul H. Spinrad A. Weiner A. Matcovitch-Natan O. Dvir-Szternfeld R. Ulland T. K.. (2017). A Unique Microglia Type Associated with Restricting Development of Alzheimer's Disease. Cell 169, 1276–1290.e17. doi: 10.1016/j.cell.2017.05.018 28602351

[ref100] Kester M. I. Teunissen C. E. Crimmins D. L. Herries E. M. Ladenson J. H. Scheltens P.. (2015). Neurogranin as a Cerebrospinal Fluid Biomarker for Synaptic Loss in Symptomatic Alzheimer Disease. JAMA Neurol. 72, 1275–1280. doi: 10.1001/jamaneurol.2015.1867 26366630 PMC4694558

[ref101] Khan T. K. Alkon D. L. (2015). Peripheral biomarkers of Alzheimer's disease. J. Alzheimers Dis. 44, 729–744. doi: 10.3233/jad-142262 25374110

[ref102] Kleinberger G. Yamanishi Y. Suárez-Calvet M. Czirr E. Lohmann E. Cuyvers E.. (2014). TREM2 mutations implicated in neurodegeneration impair cell surface transport and phagocytosis. Sci. Transl. Med. 6:243ra86. doi: 10.1126/scitranslmed.3009093 24990881

[ref103] Korff A. Liu C. Ginghina C. Shi M. Zhang J. (2013). α-Synuclein in cerebrospinal fluid of Alzheimer's disease and mild cognitive impairment. J. Alzheimers Dis. 36, 679–688. doi: 10.3233/jad-130458, PMID: 23603399 PMC3740054

[ref104] Kubota Y. Putkey J. A. Waxham M. N. (2007). Neurogranin controls the spatiotemporal pattern of postsynaptic Ca2+/CaM signaling. Biophys. J. 93, 3848–3859. doi: 10.1529/biophysj.107.106849 17704141 PMC2084249

[ref105] Kulichikhin K. Y. Fedotov S. A. Rubel M. S. Zalutskaya N. M. Zobnina A. E. Malikova O. A.. (2021). Development of molecular tools for diagnosis of Alzheimer's disease that are based on detection of amyloidogenic proteins. Prion 15, 56–69. doi: 10.1080/19336896.2021.1917289 33910450 PMC8096329

[ref106] Kvartsberg H. Portelius E. Andreasson U. Brinkmalm G. Hellwig K. Lelental N.. (2015). Characterization of the postsynaptic protein neurogranin in paired cerebrospinal fluid and plasma samples from Alzheimer's disease patients and healthy controls. Alzheimers Res. Ther. 7:40. doi: 10.1186/s13195-015-0124-3 26136856 PMC4487851

[ref107] La Joie R. Bejanin A. Fagan A. M. Ayakta N. Baker S. L. Bourakova V.. (2018). Associations between [(18)F]AV1451 tau PET and CSF measures of tau pathology in a clinical sample. Neurology 90, e282–e290. doi: 10.1212/wnl.0000000000004860 29282337 PMC5798657

[ref108] Lantero Rodriguez J. Karikari T. K. Suárez-Calvet M. Troakes C. King A. Emersic A.. (2020). Plasma p-tau181 accurately predicts Alzheimer's disease pathology at least 8 years prior to post-mortem and improves the clinical characterisation of cognitive decline. Acta Neuropathol. 140, 267–278. doi: 10.1007/s00401-020-02195-x 32720099 PMC7423866

[ref109] Lewczuk P. Esselmann H. Otto M. Maler J. M. Henkel A. W. Henkel M. K.. (2004). Neurochemical diagnosis of Alzheimer's dementia by CSF Abeta42, Abeta42/Abeta40 ratio and total tau. Neurobiol. Aging 25, 273–281. doi: 10.1016/s0197-4580(03)00086-1 15123331

[ref110] Li Y. Fang J. Zhou Z. Zhou Q. Sun S. Jin Z.. (2020). Downregulation of lncRNA BACE1-AS improves dopamine-dependent oxidative stress in rats with Parkinson's disease by upregulating microRNA-34b-5p and downregulating BACE1. Cell Cycle 19, 1158–1171. doi: 10.1080/15384101.2020.1749447 32308102 PMC7217373

[ref111] Libiger O. Shaw L. M. Watson M. H. Nairn A. C. Umaña K. L. Biarnes M. C.. (2021). Longitudinal CSF proteomics identifies NPTX2 as a prognostic biomarker of Alzheimer's disease. Alzheimers Dement. 17, 1976–1987. doi: 10.1002/alz.12353 33984181 PMC9222372

[ref112] Lill C. M. Rengmark A. Pihlstrøm L. Fogh I. Shatunov A. Sleiman P. M.. (2015). The role of TREM2 R47H as a risk factor for Alzheimer's disease, frontotemporal lobar degeneration, amyotrophic lateral sclerosis, and Parkinson's disease. Alzheimers Dement. 11, 1407–1416. doi: 10.1016/j.jalz.2014.12.009 25936935 PMC4627856

[ref113] Liu D. Dong X. Yang R. Guo H. Wang T. Xu G. (2020). Visinin-like protein-1 level is associated with short-term functional outcome of acute ischemic stroke: A prospective cohort study. Medicine 99:e19252. doi: 10.1097/md.0000000000019252 32118731 PMC7478586

[ref114] Liu J. Geraghty J. R. Schram S. Cropper H. C. Lei J. Loeb J. A.. (2023). Prevention of Alzheimer pathology by blocking neuregulin signaling on microglia. eNeuro:ENEURO.0422-23.2023:10. doi: 10.1523/eneuro.0422-23.2023 PMC10644371 37903620

[ref115] Liu W. Lin H. He X. Chen L. Dai Y. Jia W.. (2020). Neurogranin as a cognitive biomarker in cerebrospinal fluid and blood exosomes for Alzheimer's disease and mild cognitive impairment. Transl. Psychiatry 10:125. doi: 10.1038/s41398-020-0801-2 32350238 PMC7190828

[ref116] Liu P. P. Xie Y. Meng X. Y. Kang J. S. (2019). History and progress of hypotheses and clinical trials for Alzheimer's disease. Signal Transduct. Target. Ther. 4:29. doi: 10.1038/s41392-019-0063-8 31637009 PMC6799833

[ref117] Liu Q. Xie F. Siedlak S. L. Nunomura A. Honda K. Moreira P. I.. (2004). Neurofilament proteins in neurodegenerative diseases. Cell. Mol. Life Sci. 61, 3057–3075. doi: 10.1007/s00018-004-4268-8 15583867 PMC11924432

[ref118] Long J. M. Holtzman D. M. (2019). Alzheimer disease: an update on pathobiology and treatment strategies. Cell 179, 312–339. doi: 10.1016/j.cell.2019.09.001 31564456 PMC6778042

[ref119] Luo X. Hou L. Shi H. Zhong X. Zhang Y. Zheng D.. (2013). CSF levels of the neuronal injury biomarker visinin-like protein-1 in Alzheimer's disease and dementia with Lewy bodies. J. Neurochem. 127, 681–690. doi: 10.1111/jnc.12331 23800322

[ref120] Ma L. Z. Tan L. Bi Y. L. Shen X. N. Xu W. Ma Y. H.. (2020). Dynamic changes of CSF sTREM2 in preclinical Alzheimer's disease: the CABLE study. Mol. Neurodegener. 15:25. doi: 10.1186/s13024-020-00374-8 32276587 PMC7149923

[ref121] Mahaman Y. Embaye K. S. Huang F. Li L. Zhu F. Wang J. Z.. (2022). Biomarkers used in Alzheimer's disease diagnosis, treatment, and prevention. Ageing Res. Rev. 74:101544. doi: 10.1016/j.arr.2021.101544 34933129

[ref122] Malki I. Cantrelle F. X. Sottejeau Y. Lippens G. Lambert J. C. Landrieu I. (2017). Regulation of the interaction between the neuronal BIN1 isoform 1 and Tau proteins - role of the SH3 domain. FEBS J. 284, 3218–3229. doi: 10.1111/febs.14185 28755476

[ref123] Marambaud P. Dreses-Werringloer U. Vingtdeux V. (2009). Calcium signaling in neurodegeneration. Mol. Neurodegener. 4:20. doi: 10.1186/1750-1326-4-20 19419557 PMC2689218

[ref124] Mattsson N. Andreasson U. Zetterberg H. Blennow K. (2017). Association of Plasma Neurofilament Light With Neurodegeneration in Patients With Alzheimer Disease. JAMA Neurol. 74, 557–566. doi: 10.1001/jamaneurol.2016.6117 28346578 PMC5822204

[ref125] Mattsson N. Insel P. S. Palmqvist S. Portelius E. Zetterberg H. Weiner M.. (2016). Cerebrospinal fluid tau, neurogranin, and neurofilament light in Alzheimer's disease. EMBO Mol. Med. 8, 1184–1196. doi: 10.15252/emmm.201606540 27534871 PMC5048367

[ref126] Mattsson-Carlgren N. Janelidze S. Bateman R. J. Smith R. Stomrud E. Serrano G. E.. (2021). Soluble P-tau217 reflects amyloid and tau pathology and mediates the association of amyloid with tau. EMBO Mol. Med. 13:e14022. doi: 10.15252/emmm.202114022 33949133 PMC8185545

[ref127] Mavroudis I. Chowdhury R. Petridis F. Karantali E. Chatzikonstantinou S. Balmus I. M.. (2021). YKL-40 as a Potential Biomarker for the Differential Diagnosis of Alzheimer's Disease. Medicina 58:60. doi: 10.3390/medicina58010060 35056368 PMC8777884

[ref128] Mavroudis I. A. Petridis F. Chatzikonstantinou S. Karantali E. Kazis D. (2021). A meta-analysis on the levels of VILIP-1 in the CSF of Alzheimer's disease compared to normal controls and other neurodegenerative conditions. Aging Clin. Exp. Res. 33, 265–272. doi: 10.1007/s40520-019-01458-2 31939203

[ref129] Mavroudis I. A. Petridis F. Chatzikonstantinou S. Kazis D. (2020). A meta-analysis on CSF neurogranin levels for the diagnosis of Alzheimer's disease and mild cognitive impairment. Aging Clin. Exp. Res. 32, 1639–1646. doi: 10.1007/s40520-019-01326-z 31463927

[ref130] Mayeux R. Honig L. S. Tang M. X. Manly J. Stern Y. Schupf N.. (2003). Plasma A[beta]40 and A[beta]42 and Alzheimer's disease: relation to age, mortality, and risk. Neurology 61, 1185–1190. doi: 10.1212/01.wnl.0000091890.32140.8f 14610118

[ref131] McConlogue L. Buttini M. Anderson J. P. Brigham E. F. Chen K. S. Freedman S. B.. (2007). Partial reduction of BACE1 has dramatic effects on Alzheimer plaque and synaptic pathology in APP Transgenic Mice. J. Biol. Chem. 282, 26326–26334. doi: 10.1074/jbc.M611687200, PMID: 17616527

[ref132] Menendez-Gonzalez M. Gasparovic C. (2019). Albumin Exchange in Alzheimer's Disease: Might CSF Be an Alternative Route to Plasma? Front. Neurol. 10:1036. doi: 10.3389/fneur.2019.01036 31681137 PMC6813234

[ref133] Miyagawa T. Ebinuma I. Morohashi Y. Hori Y. Young Chang M. Hattori H.. (2016). BIN1 regulates BACE1 intracellular trafficking and amyloid-β production. Hum. Mol. Genet. 25, 2948–2958. doi: 10.1093/hmg/ddw146 27179792

[ref134] Montagne A. Nation D. A. Sagare A. P. Barisano G. Sweeney M. D. Chakhoyan A.. (2020). APOE4 leads to blood-brain barrier dysfunction predicting cognitive decline. Nature 581, 71–76. doi: 10.1038/s41586-020-2247-3 32376954 PMC7250000

[ref135] Montoliu-Gaya L. Alcolea D. Ashton N. J. Pegueroles J. Levin J. Bosch B.. (2023). Plasma and cerebrospinal fluid glial fibrillary acidic protein levels in adults with Down syndrome: a longitudinal cohort study. EBioMedicine 90:104547. doi: 10.1016/j.ebiom.2023.104547 37002988 PMC10070083

[ref136] Mouton-Liger F. Dumurgier J. Cognat E. Hourregue C. Zetterberg H. Vanderstichele H.. (2020). CSF levels of the BACE1 substrate NRG1 correlate with cognition in Alzheimer's disease. Alzheimers Res. Ther. 12:88. doi: 10.1186/s13195-020-00655-w 32690068 PMC7372801

[ref137] Mroczko B. Groblewska M. Zboch M. Muszyński P. Zajkowska A. Borawska R.. (2015). Evaluation of visinin-like protein 1 concentrations in the cerebrospinal fluid of patients with mild cognitive impairment as a dynamic biomarker of Alzheimer's disease. J. Alzheimers Dis. 43, 1031–1037. doi: 10.3233/jad-141050 25159667

[ref138] Nakamura A. Kaneko N. Villemagne V. L. Kato T. Doecke J. Doré V.. (2018). High performance plasma amyloid-β biomarkers for Alzheimer's disease. Nature 554, 249–254. doi: 10.1038/nature25456 29420472

[ref139] Nation D. A. Sweeney M. D. Montagne A. Sagare A. P. D'orazio L. M. Pachicano M.. (2019). Blood-brain barrier breakdown is an early biomarker of human cognitive dysfunction. Nat. Med. 25, 270–276. doi: 10.1038/s41591-018-0297-y 30643288 PMC6367058

[ref140] Nilsson J. Gobom J. Sjödin S. Brinkmalm G. Ashton N. J. Svensson J.. (2021). Cerebrospinal fluid biomarker panel for synaptic dysfunction in Alzheimer's disease. Alzheimers Dement 13:e12179. doi: 10.1002/dad2.12179 PMC8087978 33969172

[ref141] Nutu M. Zetterberg H. Londos E. Minthon L. Nägga K. Blennow K.. (2013). Evaluation of the cerebrospinal fluid amyloid-β1-42/amyloid-β1-40 ratio measured by alpha-LISA to distinguish Alzheimer's disease from other dementia disorders. Dement. Geriatr. Cogn. Disord. 36, 99–110. doi: 10.1159/000353442, PMID: 23860354

[ref142] Öhrfelt A. Brinkmalm A. Dumurgier J. Brinkmalm G. Hansson O. Zetterberg H.. (2016). The pre-synaptic vesicle protein synaptotagmin is a novel biomarker for Alzheimer's disease. Alzheimers Res. Ther. 8:41. doi: 10.1186/s13195-016-0208-8 27716408 PMC5048479

[ref143] Olsson B. Lautner R. Andreasson U. Öhrfelt A. Portelius E. Bjerke M.. (2016). CSF and blood biomarkers for the diagnosis of Alzheimer's disease: a systematic review and meta-analysis. Lancet Neurol. 15, 673–684. doi: 10.1016/s1474-4422(16)00070-3 27068280

[ref144] Ossenkoppele R. Smith R. Ohlsson T. Strandberg O. Mattsson N. Insel P. S.. (2019). Associations between tau, Aβ, and cortical thickness with cognition in Alzheimer disease. Neurology 92, e601–e612. doi: 10.1212/wnl.0000000000006875 30626656 PMC6382060

[ref145] Ovod V. Ramsey K. N. Mawuenyega K. G. Bollinger J. G. Hicks T. Schneider T.. (2017). Amyloid β concentrations and stable isotope labeling kinetics of human plasma specific to central nervous system amyloidosis. Alzheimers Dement. 13, 841–849. doi: 10.1016/j.jalz.2017.06.2266 28734653 PMC5567785

[ref146] Palmqvist S. Insel P. S. Stomrud E. Janelidze S. Zetterberg H. Brix B.. (2019). Cerebrospinal fluid and plasma biomarker trajectories with increasing amyloid deposition in Alzheimer's disease. EMBO Mol. Med. 11:e11170. doi: 10.15252/emmm.201911170 31709776 PMC6895602

[ref147] Palmqvist S. Janelidze S. Quiroz Y. T. Zetterberg H. Lopera F. Stomrud E.. (2020). Discriminative Accuracy of Plasma Phospho-tau217 for Alzheimer Disease vs Other Neurodegenerative Disorders. JAMA 324, 772–781. doi: 10.1001/jama.2020.12134 32722745 PMC7388060

[ref148] Palmqvist S. Mattsson N. Hansson O. Alzheimer’s Disease Neuroimaging Initiative (2016). Cerebrospinal fluid analysis detects cerebral amyloid-β accumulation earlier than positron emission tomography. Brain 139, 1226–1236. doi: 10.1093/brain/aww015 26936941 PMC4806222

[ref149] Pampuscenko K. Morkuniene R. Sneideris T. Smirnovas V. Budvytyte R. Valincius G.. (2020). Extracellular tau induces microglial phagocytosis of living neurons in cell cultures. J. Neurochem. 154, 316–329. doi: 10.1111/jnc.14940 31834946

[ref150] Pankonin M. S. Sohi J. Kamholz J. Loeb J. A. (2009). Differential distribution of neuregulin in human brain and spinal fluid. Brain Res. 1258, 1–11. doi: 10.1016/j.brainres.2008.12.047 19150438

[ref151] Pant S. Sharma M. Patel K. Caplan S. Carr C. M. Grant B. D. (2009). AMPH-1/Amphiphysin/Bin1 functions with RME-1/Ehd1 in endocytic recycling. Nat. Cell Biol. 11, 1399–1410. doi: 10.1038/ncb1986 19915558 PMC2788952

[ref152] Pekny M. Nilsson M. (2005). Astrocyte activation and reactive gliosis. Glia 50, 427–434. doi: 10.1002/glia.20207 15846805

[ref153] Pereira J. B. Janelidze S. Smith R. Mattsson-Carlgren N. Palmqvist S. Teunissen C. E.. (2021). Plasma GFAP is an early marker of amyloid-β but not tau pathology in Alzheimer’s disease. Brain 144, 3505–3516. doi: 10.1093/brain/awab223 34259835 PMC8677538

[ref154] Pereira J. B. Westman E. Hansson O. (2017). Association between cerebrospinal fluid and plasma neurodegeneration biomarkers with brain atrophy in Alzheimer's disease. Neurobiol. Aging 58, 14–29. doi: 10.1016/j.neurobiolaging.2017.06.002 28692877

[ref155] Piccio L. Deming Y. Del-Águila J. L. Ghezzi L. Holtzman D. M. Fagan A. M.. (2016). Cerebrospinal fluid soluble TREM2 is higher in Alzheimer disease and associated with mutation status. Acta Neuropathol. 131, 925–933. doi: 10.1007/s00401-016-1533-5 26754641 PMC4867123

[ref156] Portelius E. Olsson B. Höglund K. Cullen N. C. Kvartsberg H. Andreasson U.. (2018). Cerebrospinal fluid neurogranin concentration in neurodegeneration: relation to clinical phenotypes and neuropathology. Acta Neuropathol. 136, 363–376. doi: 10.1007/s00401-018-1851-x 29700597 PMC6096740

[ref157] Price J. L. Morris J. C. (1999). Tangles and plaques in nondemented aging and “preclinical” Alzheimer’s disease. Ann. Neurol. 45, 358–368. doi: 10.1002/1531-8249(199903)45:3<358::aid-ana12>3.0.co;2-x, PMID: 10072051

[ref158] Reitz C. Mayeux R. (2014). Alzheimer disease: epidemiology, diagnostic criteria, risk factors and biomarkers. Biochem. Pharmacol. 88, 640–651. doi: 10.1016/j.bcp.2013.12.024 24398425 PMC3992261

[ref159] Represa A. Deloulme J. C. Sensenbrenner M. Ben-Ari Y. Baudier J. (1990). Neurogranin: immunocytochemical localization of a brain-specific protein kinase C substrate. J. Neurosci. 10, 3782–3792. doi: 10.1523/jneurosci.10-12-03782.1990 2269883 PMC6570038

[ref160] Sandelius Å. Cullen N. C. Källén Å. Rosengren L. Jensen C. Kostanjevecki V.. (2018). Transient increase in CSF GAP-43 concentration after ischemic stroke. BMC Neurol. 18:202. doi: 10.1186/s12883-018-1210-5 30526557 PMC6284302

[ref161] Sandelius Å. Portelius E. Källén Å. Zetterberg H. Rot U. Olsson B.. (2019). Elevated CSF GAP-43 is Alzheimer's disease specific and associated with tau and amyloid pathology. Alzheimers Dement. 15, 55–64. doi: 10.1016/j.jalz.2018.08.006 30321501 PMC6333489

[ref162] Sartori M. Mendes T. Desai S. Lasorsa A. Herledan A. Malmanche N.. (2019). BIN1 recovers tauopathy-induced long-term memory deficits in mice and interacts with Tau through Thr(348) phosphorylation. Acta Neuropathol. 138, 631–652. doi: 10.1007/s00401-019-02017-9 31065832 PMC6778065

[ref163] Sato C. Barthélemy N. R. Mawuenyega K. G. Patterson B. W. Gordon B. A. Jockel-Balsarotti J.. (2018). Tau Kinetics in Neurons and the Human Central Nervous System. Neuron 97, 1284–1298.e7. doi: 10.1016/j.neuron.2018.02.015 29566794 PMC6137722

[ref164] Saunders T. Gunn C. Blennow K. Kvartsberg H. Zetterberg H. Shenkin S. D.. (2023). Neurogranin in Alzheimer's disease and ageing: A human post-mortem study. Neurobiol. Dis. 177:105991. doi: 10.1016/j.nbd.2023.105991 36623608

[ref165] Scheff S. W. Price D. A. Schmitt F. A. Dekosky S. T. Mufson E. J. (2007). Synaptic alterations in CA1 in mild Alzheimer disease and mild cognitive impairment. Neurology 68, 1501–1508. doi: 10.1212/01.wnl.0000260698.46517.8f 17470753

[ref166] Scheltens P. De Strooper B. Kivipelto M. Holstege H. Chételat G. Teunissen C. E.. (2021). Alzheimer's disease. Lancet 397, 1577–1590. doi: 10.1016/S0140-6736(20)32205-4 33667416 PMC8354300

[ref167] Schindler S. E. (2022). Fluid Biomarkers in Dementia Diagnosis. Continuum 28, 822–833. doi: 10.1212/con.0000000000001083 35678404

[ref168] Schindler S. E. Bollinger J. G. Ovod V. Mawuenyega K. G. Li Y. Gordon B. A.. (2019). High-precision plasma β-amyloid 42/40 predicts current and future brain amyloidosis. Neurology 93, e1647–e1659. doi: 10.1212/wnl.0000000000008081 31371569 PMC6946467

[ref169] Schneider J. A. Arvanitakis Z. Leurgans S. E. Bennett D. A. (2009). The neuropathology of probable Alzheimer disease and mild cognitive impairment. Ann. Neurol. 66, 200–208. doi: 10.1002/ana.21706 19743450 PMC2812870

[ref170] Shankar G. M. Bloodgood B. L. Townsend M. Walsh D. M. Selkoe D. J. Sabatini B. L. (2007). Natural oligomers of the Alzheimer amyloid-beta protein induce reversible synapse loss by modulating an NMDA-type glutamate receptor-dependent signaling pathway. J. Neurosci. 27, 2866–2875. doi: 10.1523/jneurosci.4970-06.2007 17360908 PMC6672572

[ref171] Shao K. Shan S. Ru W. Ma C. (2020). Association between serum NPTX2 and cognitive function in patients with vascular dementia. Brain Behav. 10:e01779. doi: 10.1002/brb3.1779 32748547 PMC7559607

[ref172] Shui B. Tao D. Florea A. Cheng J. Zhao Q. Gu Y.. (2018). Biosensors for Alzheimer's disease biomarker detection: a review. Biochimie 147, 13–24. doi: 10.1016/j.biochi.2017.12.015 29307704

[ref173] Simrén J. Elmgren A. Blennow K. Zetterberg H. (2023). Fluid biomarkers in Alzheimer's disease. Adv. Clin. Chem. 112, 249–281. doi: 10.1016/bs.acc.2022.09.006 36642485

[ref174] Simrén J. Weninger H. Brum W. S. Khalil S. Benedet A. L. Blennow K.. (2022). Differences between blood and cerebrospinal fluid glial fibrillary Acidic protein levels: The effect of sample stability. Alzheimers Dement. 18, 1988–1992. doi: 10.1002/alz.12806 36102852 PMC9826213

[ref175] Skillbäck T. Delsing L. Synnergren J. Mattsson N. Janelidze S. Nägga K.. (2017). CSF/serum albumin ratio in dementias: a cross-sectional study on 1861 patients. Neurobiol. Aging 59, 1–9. doi: 10.1016/j.neurobiolaging.2017.06.028 28779628

[ref176] Skillbäck T. Rosén C. Asztely F. Mattsson N. Blennow K. Zetterberg H. (2014). Diagnostic performance of cerebrospinal fluid total tau and phosphorylated tau in Creutzfeldt-Jakob disease: results from the Swedish Mortality Registry. JAMA Neurol. 71, 476–483. doi: 10.1001/jamaneurol.2013.6455 24566866

[ref177] Smirnov D. S. Ashton N. J. Blennow K. Zetterberg H. Simrén J. Lantero-Rodriguez J.. (2022). Plasma biomarkers for Alzheimer's Disease in relation to neuropathology and cognitive change. Acta Neuropathol. 143, 487–503. doi: 10.1007/s00401-022-02408-5 35195758 PMC8960664

[ref178] Srivastava S. Ahmad R. Khare S. K. (2021). Alzheimer's disease and its treatment by different approaches: a review. Eur. J. Med. Chem. 216:113320. doi: 10.1016/j.ejmech.2021.113320 33652356

[ref179] Stejskal D. Sporova L. Svestak M. Karpisek M. (2011). Determination of serum visinin like protein-1 and its potential for the diagnosis of brain injury due to the stroke: a pilot study. Biomed. Pap. Med. Fac. Univ. Palacky Olomouc Czech Repub. 155, 263–268. doi: 10.5507/bp.2011.049 22286812

[ref180] Stomrud E. Hansson O. Blennow K. Minthon L. Londos E. (2007). Cerebrospinal fluid biomarkers predict decline in subjective cognitive function over 3 years in healthy elderly. Dement. Geriatr. Cogn. Disord. 24, 118–124. doi: 10.1159/000105017 17622715

[ref181] Strozyk D. Blennow K. White L. R. Launer L. J. (2003). CSF Abeta 42 levels correlate with amyloid-neuropathology in a population-based autopsy study. Neurology 60, 652–656. doi: 10.1212/01.wnl.0000046581.81650.d0 12601108

[ref182] Suárez-Calvet M. Araque Caballero M. Kleinberger G. Bateman R. J. Fagan A. M. Morris J. C.. (2016a). Early changes in CSF sTREM2 in dominantly inherited Alzheimer's disease occur after amyloid deposition and neuronal injury. Sci. Transl. Med. 8:369ra178. doi: 10.1126/scitranslmed.aag1767 PMC5385711 27974666

[ref183] Suárez-Calvet M. Karikari T. K. Ashton N. J. Lantero Rodríguez J. Milà-Alomà M. Gispert J. D.. (2020). Novel tau biomarkers phosphorylated at T181, T217 or T231 rise in the initial stages of the preclinical Alzheimer's continuum when only subtle changes in Aβ pathology are detected. EMBO Mol. Med. 12:e12921. doi: 10.15252/emmm.202012921 33169916 PMC7721364

[ref184] Suárez-Calvet M. Kleinberger G. Araque Caballero M. Brendel M. Rominger A. Alcolea D.. (2016b). sTREM2 cerebrospinal fluid levels are a potential biomarker for microglia activity in early-stage Alzheimer's disease and associate with neuronal injury markers. EMBO Mol. Med. 8, 466–476. doi: 10.15252/emmm.201506123 26941262 PMC5120370

[ref185] Sudwarts A. Ramesha S. Gao T. Ponnusamy M. Wang S. Hansen M.. (2022). BIN1 is a key regulator of proinflammatory and neurodegeneration-related activation in microglia. Mol. Neurodegener. 17:33. doi: 10.1186/s13024-022-00535-x 35526014 PMC9077874

[ref186] Sun L. Tan M. S. Hu N. Yu J. T. Tan L. (2013). Exploring the value of plasma BIN1 as a potential biomarker for alzheimer's disease. J. Alzheimers Dis. 37, 291–295. doi: 10.3233/jad-130392 23803295

[ref187] Sunderland T. Linker G. Mirza N. Putnam K. T. Friedman D. L. Kimmel L. H.. (2003). Decreased beta-amyloid1-42 and increased tau levels in cerebrospinal fluid of patients with Alzheimer disease. JAMA 289, 2094–2103. doi: 10.1001/jama.289.16.2094 12709467

[ref188] Sutphen C. L. Mccue L. Herries E. M. Xiong C. Ladenson J. H. Holtzman D. M.. (2018). Longitudinal decreases in multiple cerebrospinal fluid biomarkers of neuronal injury in symptomatic late onset Alzheimer's disease. Alzheimers Dement. 14, 869–879. doi: 10.1016/j.jalz.2018.01.012 29580670 PMC6110083

[ref189] Tan Z. Jiang J. Tian F. Peng J. Yang Z. Li S.. (2020). Serum Visinin-Like Protein 1 Is a Better Biomarker Than Neuron-Specific Enolase for Seizure-Induced Neuronal Injury: A Prospective and Observational Study. Front. Neurol. 11:567587. doi: 10.3389/fneur.2020.567587 33071949 PMC7544981

[ref190] Tan M. S. Yu J. T. Tan L. (2013). Bridging integrator 1 (BIN1): form, function, and Alzheimer's disease. Trends Mol. Med. 19, 594–603. doi: 10.1016/j.molmed.2013.06.004 23871436

[ref191] Tarasoff-Conway J. M. Carare R. O. Osorio R. S. Glodzik L. Butler T. Fieremans E.. (2015). Clearance systems in the brain-implications for Alzheimer disease. Nat. Rev. Neurol. 11, 457–470. doi: 10.1038/nrneurol.2015.119 26195256 PMC4694579

[ref192] Teunissen C. E. Verberk I. M. W. Thijssen E. H. Vermunt L. Hansson O. Zetterberg H.. (2022). Blood-based biomarkers for Alzheimer's disease: towards clinical implementation. Lancet Neurol. 21, 66–77. doi: 10.1016/s1474-4422(21)00361-6 34838239

[ref193] Thijssen E. H. La Joie R. Strom A. Fonseca C. Iaccarino L. Wolf A.. (2021). Plasma phosphorylated tau 217 and phosphorylated tau 181 as biomarkers in Alzheimer's disease and frontotemporal lobar degeneration: a retrospective diagnostic performance study. Lancet Neurol. 20, 739–752. doi: 10.1016/s1474-4422(21)00214-3 34418401 PMC8711249

[ref194] Thijssen E. H. La Joie R. Wolf A. Strom A. Wang P. Iaccarino L.. (2020). Diagnostic value of plasma phosphorylated tau181 in Alzheimer's disease and frontotemporal lobar degeneration. Nat. Med. 26, 387–397. doi: 10.1038/s41591-020-0762-2 32123386 PMC7101073

[ref195] Thomas S. Hoxha K. Tran A. Prendergast G. C. (2019). Bin1 antibody lowers the expression of phosphorylated Tau in Alzheimer's disease. J. Cell. Biochem. 120, 18320–18331. doi: 10.1002/jcb.29142 31211444

[ref196] Tibbling G. Link H. Ohman S. (1977). Principles of albumin and IgG analyses in neurological disorders. I. Establishment of reference values. Scand. J. Clin. Lab. Invest. 37, 385–390. doi: 10.1080/00365517709091496 337459

[ref197] Uberti D. Cantarella G. Facchetti F. Cafici A. Grasso G. Bernardini R.. (2004). TRAIL is expressed in the brain cells of Alzheimer's disease patients. Neuroreport 15, 579–581. doi: 10.1097/00001756-200403220-00002 15094456

[ref198] Uberti D. Ferrari-Toninelli G. Bonini S. A. Sarnico I. Benarese M. Pizzi M.. (2007). Blockade of the tumor necrosis factor-related apoptosis inducing ligand death receptor DR5 prevents beta-amyloid neurotoxicity. Neuropsychopharmacology 32, 872–880. doi: 10.1038/sj.npp.1301185 16936710

[ref199] Ueno M. Chiba Y. Murakami R. Matsumoto K. Kawauchi M. Fujihara R. (2016). Blood-brain barrier and blood-cerebrospinal fluid barrier in normal and pathological conditions. Brain Tumor Pathol. 33, 89–96. doi: 10.1007/s10014-016-0255-7 26920424

[ref200] Vassar R. Bennett B. D. Babu-Khan S. Kahn S. Mendiaz E. A. Denis P.. (1999). Beta-secretase cleavage of Alzheimer's amyloid precursor protein by the transmembrane aspartic protease BACE. Science 286, 735–741. doi: 10.1126/science.286.5440.735 10531052

[ref201] Verberk I. M. W. Thijssen E. Koelewijn J. Mauroo K. Vanbrabant J. De Wilde A.. (2020). Combination of plasma amyloid beta((1-42/1-40)) and glial fibrillary acidic protein strongly associates with cerebral amyloid pathology. Alzheimers Res. Ther. 12:118. doi: 10.1186/s13195-020-00682-7 32988409 PMC7523295

[ref202] Vermunt L. Sikkes S. van den Hout A. Handels R. Bos I. van der Flier W. M.. (2019). Duration of preclinical, prodromal, and dementia stages of Alzheimer's disease in relation to age, sex, and APOE genotype. Alzheimers Dement. 15, 888–898. doi: 10.1016/j.jalz.2019.04.001 31164314 PMC6646097

[ref203] Villar-Piqué A. Schmitz M. Hermann P. Goebel S. Bunck T. Varges D.. (2019). Plasma YKL-40 in the spectrum of neurodegenerative dementia. J. Neuroinflammation 16:145. doi: 10.1186/s12974-019-1531-3 31299989 PMC6624942

[ref204] Visser P. J. Verhey F. Knol D. L. Scheltens P. Wahlund L. O. Freund-Levi Y.. (2009). Prevalence and prognostic value of CSF markers of Alzheimer's disease pathology in patients with subjective cognitive impairment or mild cognitive impairment in the DESCRIPA study: a prospective cohort study. Lancet Neurol. 8, 619–627. doi: 10.1016/s1474-4422(09)70139-5 19523877

[ref205] Vrillon A. Mouton-Liger F. Martinet M. Cognat E. Hourregue C. Dumurgier J.. (2022). Plasma neuregulin 1 as a synaptic biomarker in Alzheimer's disease: a discovery cohort study. Alzheimers Res. Ther. 14:71. doi: 10.1186/s13195-022-01014-7 35606871 PMC9125890

[ref206] Wagner O. I. Rammensee S. Korde N. Wen Q. Leterrier J. F. Janmey P. A. (2007). Softness, strength and self-repair in intermediate filament networks. Exp. Cell Res. 313, 2228–2235. doi: 10.1016/j.yexcr.2007.04.025 17524395 PMC2709732

[ref207] Wallin Å. K. Blennow K. Zetterberg H. Londos E. Minthon L. Hansson O. (2010). CSF biomarkers predict a more malignant outcome in Alzheimer disease. Neurology 74, 1531–1537. doi: 10.1212/WNL.0b013e3181dd4dd8 20458070

[ref208] Wang Z. Y. Han Z. M. Liu Q. F. Tang W. Ye K. Yao Y. Y. (2015). Use of CSF α-synuclein in the differential diagnosis between Alzheimer’s disease and other neurodegenerative disorders. Int. Psychogeriatr. 27, 1429–1438. doi: 10.1017/s1041610215000447 25851548

[ref209] Wang J. Qiao F. Shang S. Li P. Chen C. Dang L.. (2018). Elevation of Plasma Amyloid-β Level is More Significant in Early Stage of Cognitive Impairment: A Population-Based Cross-Sectional Study. J. Alzheimers Dis. 64, 61–69. doi: 10.3233/jad-180140 29865072

[ref210] Wang Z. Tan L. Zong Y. Ma Y. H. Wang Z. B. Wang H. F.. (2023). sTREM2 and GFAP Mediated the Association of IGF-1 Signaling Biomarkers with Alzheimer's Disease Pathology. J. Alzheimers Dis. 92, 791–797. doi: 10.3233/jad-220725 36806504

[ref211] Wang S. Zhang J. Pan T. (2018). APOE ε4 is associated with higher levels of CSF SNAP-25 in prodromal Alzheimer's disease. Neurosci. Lett. 685, 109–113. doi: 10.1016/j.neulet.2018.08.029 30144541

[ref212] Wang L. Zhang M. Wang Q. Jiang X. Li K. Liu J. (2020). APOE ε4 Allele Is Associated with Elevated Levels of CSF VILIP-1 in Preclinical Alzheimer's Disease. Neuropsychiatr. Dis. Treat. 16, 923–931. doi: 10.2147/ndt.S235395 32308396 PMC7156263

[ref213] Wechsler-Reya R. Elliott K. Prendergast G. (1998). A Role for the Putative Tumor Suppressor Bin1 in Muscle Cell Differentiation. Mol. Cell. Biol. 18, 566–575. doi: 10.1128/MCB.18.1.566 9418903 PMC121524

[ref214] Wennström M. Surova Y. Hall S. Nilsson C. Minthon L. Hansson O.. (2015). The Inflammatory Marker YKL-40 Is Elevated in Cerebrospinal Fluid from Patients with Alzheimer's but Not Parkinson's Disease or Dementia with Lewy Bodies. PLoS One 10:e0135458. doi: 10.1371/journal.pone.0135458 26270969 PMC4536228

[ref215] Weston P. S. J. Poole T. Ryan N. S. Nair A. Liang Y. Macpherson K.. (2017). Serum neurofilament light in familial Alzheimer disease: A marker of early neurodegeneration. Neurology 89, 2167–2175. doi: 10.1212/wnl.0000000000004667 29070659 PMC5696646

[ref216] Willemse E. Sieben A. Somers C. Vermeiren Y. De Roeck N. Timmers M.. (2021). Neurogranin as biomarker in CSF is non-specific to Alzheimer’s disease dementia. Neurobiol. Aging 108, 99–109. doi: 10.1016/j.neurobiolaging.2021.08.002 34551375

[ref217] Winston C. N. Goetzl E. J. Akers J. C. Carter B. S. Rockenstein E. M. Galasko D.. (2016). Prediction of conversion from mild cognitive impairment to dementia with neuronally derived blood exosome protein profile. Alzheimers Dement 3, 63–72. doi: 10.1016/j.dadm.2016.04.001 PMC4925777 27408937

[ref218] Wojdała A. L. Bellomo G. Gaetani L. Toja A. Chipi E. Shan D.. (2023). Trajectories of CSF and plasma biomarkers across Alzheimer's disease continuum: disease staging by NF-L, p-tau181, and GFAP. Neurobiol. Dis. 189:106356. doi: 10.1016/j.nbd.2023.106356 37977432

[ref219] World Health Organization (2017). Global action plan on the public health response to dementia 2017–2025. (Geneva: World Health Organization).

[ref220] Wu Y. Y. Hsu J. L. Wang H. C. Wu S. J. Hong C. J. Cheng I. H. (2015). Alterations of the neuroinflammatory markers IL-6 and TRAIL in Alzheimer’s disease. Dement. Geriatr. Cogn. Dis. Extra 5, 424–434. doi: 10.1159/000439214 26675645 PMC4677720

[ref221] Xiao M. F. Xu D. Craig M. T. Pelkey K. A. Chien C. C. Shi Y.. (2017). NPTX2 and cognitive dysfunction in Alzheimer’s Disease. Elife 6:e23798. doi: 10.7554/eLife.23798 28440221 PMC5404919

[ref222] Xie X.-Y. Zhao Q.-H. Huang Q. Dammer E. Chen S.-D. Ren R.-J.. (2022). Genetic profiles of familial late-onset Alzheimer’s disease in China: the Shanghai FLOAD study. Genes Diseases 9, 1639–1649. doi: 10.1016/j.gendis.2021.05.001 36157508 PMC9485165

[ref223] Yan Y. Jensen K. Brown A. (2007). The polypeptide composition of moving and stationary neurofilaments in cultured sympathetic neurons. Cell Motil. Cytoskeleton 64, 299–309. doi: 10.1002/cm.20184 17285620 PMC1978456

[ref224] Yang L. B. Lindholm K. Yan R. Citron M. Xia W. Yang X. L.. (2003). Elevated beta-secretase expression and enzymatic activity detected in sporadic Alzheimer disease. Nat. Med. 9, 3–4. doi: 10.1038/nm0103-3 12514700

[ref225] Yin Z. Raj D. Saiepour N. Van Dam D. Brouwer N. Holtman I. R.. (2017). Immune hyperreactivity of Aβ plaque-associated microglia in Alzheimer’s disease. Neurobiol. Aging 55, 115–122. doi: 10.1016/j.neurobiolaging.2017.03.021 28434692

[ref226] Zetterberg H. Andreasson U. Hansson O. Wu G. Sankaranarayanan S. Andersson M. E.. (2008). Elevated cerebrospinal fluid BACE1 activity in incipient Alzheimer disease. Arch. Neurol. 65, 1102–1107. doi: 10.1001/archneur.65.8.1102 18695061

[ref227] Zetterberg H. Hietala M. A. Jonsson M. Andreasen N. Styrud E. Karlsson I.. (2006). Neurochemical aftermath of amateur boxing. Arch. Neurol. 63, 1277–1280. doi: 10.1001/archneur.63.9.1277 16966505

[ref228] Zetterberg H. Skillbäck T. Mattsson N. Trojanowski J. Q. Portelius E. Shaw L. M.. (2016). Association of cerebrospinal fluid neurofilament light concentration with Alzheimer disease progression. JAMA Neurol. 73, 60–67. doi: 10.1001/jamaneurol.2015.3037 26524180 PMC5624219

[ref229] Zhang X. Tang L. Yang J. Meng L. Chen J. Zhou L.. (2023). Soluble TREM2 ameliorates tau phosphorylation and cognitive deficits through activating transgelin-2 in Alzheimer's disease. Nat. Commun. 14:6670. doi: 10.1038/s41467-023-42505-x 37865646 PMC10590452

[ref230] Zhang H. Therriault J. Kang M. S. Ng K. P. Pascoal T. A. Rosa-Neto P.. (2018). Cerebrospinal fluid synaptosomal-associated protein 25 is a key player in synaptic degeneration in mild cognitive impairment and Alzheimer’s disease. Alzheimers Res. Ther. 10:80. doi: 10.1186/s13195-018-0407-6 30115118 PMC6097333

[ref231] Zhao A. Jiao Y. Ye G. Kang W. Tan L. Li Y.. (2022). Soluble TREM2 levels associate with conversion from mild cognitive impairment to Alzheimer’s disease. J. Clin. Invest. 132:e158708. doi: 10.1172/jci158708 36519540 PMC9753995

[ref232] Zhou S. Li Y. Zhang Z. Yuan Y. (2023). An insight into the TAM system in Alzheimer's disease. Int. Immunopharmacol. 116:109791. doi: 10.1016/j.intimp.2023.109791 36738678

[ref233] Zhou J. Wade S. D. Graykowski D. Xiao M. F. Zhao B. Giannini L. A. A.. (2023). The neuronal pentraxin Nptx2 regulates complement activity and restrains microglia-mediated synapse loss in neurodegeneration. Sci. Transl. Med. 15:eadf0141. doi: 10.1126/scitranslmed.adf0141 36989373 PMC10467038

[ref234] Zhou W. Zhou Y. Li J. (2023). Association between cerebrospinal fluid soluble TREM2, Alzheimer’s disease and other neurodegenerative diseases. J. Clin. Med. 12:3589. doi: 10.3390/jcm12103589 37240695 PMC10219449

[ref235] Zhu Y. Guo X. Zhu F. Zhang Q. Yang Y. For The Alzheimer’s Disease Neuroimaging Initiative (2023). Association of CSF GAP-43 and APOE ε4 with Cognition in Mild Cognitive Impairment and Alzheimer's Disease. Cells 12:13. doi: 10.3390/cells12010013, PMID: 36611808 PMC9818551

[ref236] Zuliani G. Trentini A. Rosta V. Guerrini R. Pacifico S. Bonazzi S.. (2020). Increased blood BACE1 activity as a potential common pathogenic factor of vascular dementia and late onset Alzheimer’s disease. Sci. Rep. 10:14980. doi: 10.1038/s41598-020-72168-3 32917964 PMC7486910

